# Supplementary material for: Mitochondrial dysfunction characterises the multigenerational effects of maternal obesity on MASLD
Source: JHEP Rep. 2025 Mar 29;7(6):101404. doi: 10.1016/j.jhepr.2025.101404 (PMC12151218; doi:10.1016/j.jhepr.2025.101404)
Supplement: Multimedia component 1 [file mmc1.pdf]

Mitochondrial dysfunction characterises the multigenerational effects of maternal obesity on MASLD

Anneleen Heldens, Milton Antwi, Louis Onghena, Tim Meese, Yannick Gansemans, Joél Smet, Ellen Dupont, Xavier Verhelst, Sarah Raevens, Hans Van Vlierberghe, Arnaud Vanlander, Filip Van Nieuwerburgh, Lindsey Devisscher, Ruth De Bruyne, Anja Geerts, Sander Lefere

Table of contents

Supplementary materials and methods……………………………………………..2

Supplementary tables...12

Supplementary figures 14

Supplementary references 21

Supplementary materials and methods

Tissue sampling

Before sacrifice, mice were anesthetized with xylazine (10mg/kg; Sedaxyl) and ketamine (100mg/kg; Nimatek). After taken blood samples retro-orbitally, mice were euthanized via cervical dislocation. Liver, spleen and gonadal adipose tissue were weighed. Specific liver lobes were isolated for histology, gene expression analysis and flow cytometry.

Histology

Part of the liver were fixed in 4% paraformaldehyde (VWR, Avantor, Leuven, Belgium) for 24h and subsequently embedded in paraffin. Tissues were sectioned at 4µm using a Leica RM2145 microtome (Leica Biosystems, Diegem, Belgium). Liver sections were stained with hematoxylin-eosin (H&E) (Sigma-Aldrich, Overijse, Belgium) and Sirius red (SR) (Sigma-Aldrich) to evaluate MASLD severity and fibrosis respectively. MASLD severity was assessed according to the NAFLD activity score which considers steatosis, hepatocyte ballooning and lobular inflammation. Additionally, Sirius red area was evaluated according to the NAFLD clinical research network fibrosis scoring system and quantified using ImageJ software.

Serum analysis

Blood samples were centrifuged at 4°C. Serum was stored at -80°C before determining alanine aminotransferase (ALT), uric acid and glucose levels (UV test at 37°C; Roche Modular pre-analytics system, Rotkreuz, Switzerland). Serum insulin

levels were determined by enzyme-linked immunosorbent assay (EZRMI-13K; Merck, Overijse, Belgium) according to the manufacturer's instructions.

Intraperitoneal glucose tolerance test

An intraperitoneal glucose tolerance test (IPGTT) was performed seven days before sacrifice. Mice were fasted for 5h prior to intraperitoneal injection of glucose (2g/kg dissolved in 0.9% NaCl; Sigma-Aldrich). Blood glucose was measured in the tail vein with a glucometer (Bayer Contour Next, Basel, Switzerland) at baseline and 30, 60 and 120 min after glucose injection.

Flow cytometry and fluorescence activated cell sorting

The left liver lobe was perfused with cold phosphate buffered saline (PBS; Gibco, Thermo Fisher Scientific, Merelbeke, Belgium) and dissociated enzymatically through incubation with 1mg/mL collagenase A (Sigma-Aldrich) and 300µg/mL DNAse I (Sigma-Aldrich) at 37°C, and mechanically using the gentleMACS dissociator (Miltenyi Biotec, Leiden, The Netherlands). Prestaining of the single cell suspension was performed using Zombie Aqua (Fixable Viability Dye; Biolegend, London, United Kingdom), Trustain FcX Plus (anti-mouse CD16/32) and True-Stain monocyte blocker (Biolegend). Subsequently, cells were stained with CD31-PE/Dazzle 594, Clec4F-AF647, F4/80-FITC, Ly6C-BV650, Ly6G-BV785 (Biolegend), CD11b-PE-Cy7, CD45-APC-Cy7, SiglecF-PerCP-Cy5.5 and Tim4-PE (BD Biosciences, Erembodegem, Belgium) and analyzed with a BD FACSAria Fusion flow cytometer (BD Biosciences) and FlowJo software (FlowJo LLC, BD Biosciences). Absolute cell numbers were calculated using Precision Count Beads (Biolegend). After selection of live $CD45^+$ single cells, neutrophils were gated as $Ly6G^+$, monocytes as $Ly6G^-$ $CD11b^+$ $Ly6C^{hi}$,

MoKCs as Ly6G$^-$ CD11b$^+$ Ly6C^{lo} Clec4F$^+$ Tim4$^-$, KCs as Ly6G$^-$ CD11b$^+$ Ly6C^{lo} Clec4F$^+$ Tim4$^+$, and MoMfs as Ly6G$^-$ CD11b$^+$ Ly6C^{lo} Clec4F$^-$ Tim4$^-$ F4/80$^+$ SiglecF$^-$ SSC^{lo}.

Hepatic triglyceride assay

Snapfrozen liver tissue was homogenized in IGEPAL CA-360 (Sigma-Aldrich) using the TissueLyser LT (Qiagen, Hilden, Germany). Shaking was performed at 50Hz for 1 and subsequently 2 minutes. Triglyceride content was measured using the Triglyceride quantification kit (MAK266; Sigma-Aldrich) according to the manufacturer's instructions. The absorbance was determined at 570nm using Ascent software version 2.6 (Thermo Fisher Scientific).

Genotyping LIRKO mice

The genotype of breeding mice and male offspring was determined on tail or ear biopsies. DNA was extracted by boiling the tissue sample in PCR buffer (25mM NaOH and 0.2mM EDTA in distilled water; pH = 8). PCR was performed using the Biotaq DNA polymerase kit (Bioline, London, United Kingdom), dNTP mix (bioline) and the following primers: forward insulinreceptor floxed primer 5'-GGGGCAGTCAGTATTTTGGA-3', Reverse insulin receptor floxed primer 5'-TGGCCGTGAAAGTTAAGAGG-3'. For determining Cre genotype, the following primers were used: 5'-CGCCGTAAATCAATCGATGAGTTGCTTS-3', 5'-GATGCCGGTGAACGTGCAAAACAGGCTC-3', 5'-CAAACCTGCTACCCGAACCT-3', and 5'-CAGTATGCGGAAGTTCTAGG-3'. This results in a 145bp band for IR floxed mice and a 105bp band for wild type mice. The presence of the Cre allele results in a 500bp band.

RNA isolation and quantitative PCR (qPCR)

After homogenization of 20mg full liver tissue with the TissueLyser LT (Qiagen) as described above, RNA was extracted using the Aurum Total RNA Mini Kit (Bio-rad Laboratories, Temse, Belgium) according to the manufacturer's instructions. RNA concentration and purity was measured by spectrophotometry (Nanodrop ND-1000; Thermo Fisher Scientific). cDNA was obtained using the SensiFast cDNA Synthesis Kit (Bioline) according to the manufacturer's instructions, after dilution of RNA samples to a concentration of 100ng/µL with RNase-free water. Analysis was performed on the LightCycler 480 (Roche, Vilvoorde, Belgium) using diluted cDNA (1/10), SYBR Green mix (Sensimix; Bioline) and specific mouse primers (Bioline) (Table S1). Samples were measured in duplicate. Reactions were normalized with the following household genes: *Hmbs*, *Hprt* and *Gapdh*. Cq values were calculated with the second derivative maximum method.

RNA sequencing

Full liver tissue from the following 16-week-old offspring was sequenced: NC-fed (n = 5) and WD-fed (n = 5) offspring without maternal obesity and WD-fed offspring with maternal obesity with (n = 3) and without (n = 5) fibrosis development. RNA integrity of the samples was checked using the RNA 6000 Pico Kit on a Bioanalyer (Agilent Technologies Inc, Santa Clara, CA, USA). Concentration of input RNA was measured using the Quant-iT RiboGreen RNA Assay Kit (Invitrogen Inc, Waltham, MA, USA). A sequencing library was constructed for each sample using 500 ng of input RNA with the QuantSeq 3' mRNA-Seq Library Prep Kit FWD for Illumina (Lexogen Inc, Greenland, NH, USA) and the UMI Second Strand Synthesis Module for QuantSeq FWD (Illumina Inc, San Diego, CA, USA) to incorporate a 6 nt unique molecular

identifier (UMI) present at the 5'-end of the read after sequencing. Library enrichment was done with 13 PCR cycles and clean-up was done using the kit's PB beads. Quality control of the libraries was done on a Bioanalyzer using a High Sensitivity DNA Kit (Agilent Technologies Inc, Santa Clara, CA, USA) and via qPCR according to the Illumina Sequencing Library qPCR Quantification Guide. Finally, the libraries were spiked with 20% PhiX (Illumina Inc, San Diego, CA, USA) and sequenced as single-read 76 on a NextSeq 500 device (Illumina, San Diego, CA, USA).

<u>Differential expression analysis of RNAseq data</u>

Quality control of the raw sequencing reads was done with FastQC (v0.11.9). Contamination was checked using FastQ Screen (v0.15.1) and genomes from a limited set of common lab organisms. We used UMI-tools (v1.1.2) to remove the Unique molecular identifier (UMI) from the read sequence and add it to the read name. Adapter and quality trimming was done with cutadapt (v4.1) using default parameters with the additional use of a phred score threshold of 20. The trimmed reads were mapped on the mouse genome (GRCm39, ENSEMBL release 104) using the splice aware STAR (v2.7.10a) mapper and sorted using samtools (v1.6). Removal of mapped PCR duplicated reads was done with UMI-tools (v1.1.2), using the UMI and mapping information. Expression counts at the gene level were generated with rsem-calculate-expression (RSEM v1.36.1). To estimate if sequencing depth was reflecting the sample's complexity, we collected data for saturation plots using preseq (v3.1.1). Additional QC was done using RseQC (v4.0.0). All statistical analyses were done in R (v4.2.1) using the DESeq2's (v1.36.0) package for differential expression analysis. Multiple testing correction of p values was done using the Benjamini-Hochberg method. Differentially expressed features were considered significant when having a

fold change reflecting at least a doubling or halving (i.e., the absolute value of log2FC ≥ 1), and a corrected p value ≤ 0.05. Data were deposited to the NCBI GEO database under the accession number GSE291363.

Differential expression analysis of NCBI GEO RNAseq data from non-human primate liver samples

Raw sequencing reads, experimental design and sample metadata were downloaded from NCBI GEO accession GSE220102. The data set consisted of 33 samples in 4 treatment groups.

Raw sequencing reads were inspected using FastQC (v0.12.1) for their quality and length. Putative contaminations were checked using FastQ Screen (v0.15.3) and a set of genomes of common lab organisms. Adaptor trimming was done using cutadapt (v4.4) with added filtering of reads containing ambiguities or not passing the phred score threshold of 20. For each sample, trimmed reads were mapped on the Macaca mulatta genome (Macaca_mulatta.Mmul_10, ENSEMBL release 109) using the splice-aware STAR (v2.7.10a) mapper. UMI-based deduplication of mapped reads was done with UMI-tools (v1.1.4). Feature counting at the gene and transcript isoform level was done using rsem-calculate-expression (RSEM v1.3.3). All statistical analyses were done in R (v4.2.2) and pairwise comparisons of treatments were done using the edgeR (v3.38.4) package. Correction of the p values for repeated testing (PAdj) was done with the Benjamini-Hochberg method. Differentially expressed features were considered significant when having a fold change reflecting at least a doubling or halving (i.e., the absolute value of log2FC ≥1), and a corrected p value ≤0.05. Pathway enrichment analysis was done in R using the GAGE package (v2.44.0)

with KEGG Macaca mulatta pathway data. GO term enrichment analysis was also done with the GAGE package.

Blue-native polyacrylamide gel-electrophoresis (BN-PAGE) and in-gel activity staining of OXPHOS complexes

Mitochondria were isolated from 50mg of liver tissue. First, liver tissue with 19 volumes of ice cold mitochondrial isolation buffer (10mM Tris-HCl, 0.25M sucrose, 2mM EDTA and 50U/mL heparin, pH 7.4) was minced using scissors. Homogenization of the tissue was performed using a motor driven glass pestle with application of 20 strokes at 800rpm. Subsequently, the homogenates were sonicated for 2 seconds at medium intensity and centrifugated at 5600g for 2 minutes. The supernatant with mitochondria was kept on ice, while the pellet with remaining mitochondria was resuspended with mitochondrial isolation buffer and the procedure was repeated twice. The combined supernatant was centrifuged at 37,500g for 4 minutes and the resulting pellet was homogenized with 1.0mL of mitochondrial isolation buffer using a glass/glass pestle by applying 10 strokes. After centrifugating at 5600g for 45 seconds, the supernatant was centrifuged again at 16,100g for 15 minutes resulting in the pellet containing the mitochondria. This pellet was stored at -80°C until further analysis.

For BN-PAGE analysis, the mitochondrial pellets were resuspended in 130-170µL of 750mM aminocaproic acid, 50mM Bis-Tris/HCl (pH 7.0) and 1.1% laurylmaltoside. After centrifugation at 16,100g for 15 minutes, the supernatants containing the oxidative phosphorylation enzyme complexes were kept on ice. Protein concentration of the mitochondrial proteins was measured using the Pierce Coomassie (Bradford) Protein assay Kit (Thermo Fisher Scientific cat. 23200).

Prior to loading on the Blue Native gel, a loading dye was added to the mixture and 5µL of 5% Serva Blue G and 750mM aminocaproic acid solution was added for each 100µL of solubilized protein solution with subsequently vortexing. Electrophoresis is first performed with the colored cathodal buffer (50mM Tris and 15mM Bis-Tris and 0.02% Serva Blue G) and anodal (25mM Bis-Tris) buffer at 80V for 1.5h at 4°C, until the dye-front reaches the resolving gel. Then, the colored cathodal buffer is replaced by the non-colored cathodal buffer (50mM Tris and 15mM Bis-Tris) and run at 200V for 2-3h at 4°C, until the Serva Blue tracking dye runs off the gel. Gels are stored between glass plates at -80°C until in-gel activity staining.

Samples were run in duplicate on two separate BN PAGE gels. The first gel was divided in three pieces: the upper, middle and lower part was used to evaluate complex I, complex III and complex IV activity, respectively. The second gel was divided in two pieces: the upper and lower part to evaluate the complex V and complex II activity, respectively. Complex I enzyme activity was evaluated after incubation of the gel part in a medium containing 2mM Tris-HCl 0.3mM NADH, 3.5mM NBT (pH 7.4) at 37°C for 3-4h. Complex II enzyme activity was evaluated after incubation in a medium containing 4.5mM EDTA, 10mM KCN, 0.2 mM phenazine methosulfate, 84mM sodium succinate and 10mM Nitro Blue tetrazolium chloride in 1.5mM phosphate buffer (pH 7.4) at 37°C for 3-4h. Staining of complex III was obtained by incubation with the 1-Step_TMB-Blotting Substrate Solution (Thermo Fisher Scientific) at 37°C, with maximum intensity of the bands after 5-6h. Staining of complex IV was obtained by incubation in 50mM phosphate buffer (pH 7.4), containing 2.3mM 3,3'-diaminobenzidine, 2mg/mL catalase, 1mg/mL cytochrome c and 220mM sucrose (pH 7.4) at 37°C, with maximum intensity of the bands after 5-6h. The activity of complex V was evaluated after incubation in 35mM Tris, 270mM glycine, 14mM $MgSO_4$, 0.2%

$Pb(NO_3)_2$, and 8mM ATP (pH 7.8) at 37°C, with maximum intensity of the bands after 2-3h. Gels were scanned using an Epson Perfection V800 Photo Scanner with Silverfast software in reflection mode (complex V) and in transmission mode (complex I-IV).

Mitochondrial DNA copy number

After homogenization of full liver tissue using the TissueLyser as described above, total DNA was isolated using DNeasy blood and tissue kit (Qiagen) according to the manufacturer's instructions. DNA concentrations were measured by spectrophotometry (Nanodrop ND-1000; Thermo Fisher Scientific). Analysis was performed on the LightCycler 480 (Roche) using DNA, SYBR Green mix (sensimix; Bioline) and specific mouse primers (Bioline) (Table S2).

Patients

Serum samples were collected from adolescents with severe obesity residing at the Zeepreventorium De Haan, and from lean controls. An informed consent was signed by all patients, controls and parents before enrollment. The study was approved by the ethical committee of Ghent University Hospital (BS-05660). Patients and controls underwent anthropometric measurements, serum biochemical analysis, ultrasound and FibroScan.

Liver steatosis and fibrosis were evaluated using ultrasound and FibroScan with controlled attenuation parameter (CAP) and liver stiffness measurement (LSM). Both were performed by the same experienced operator (S.L.) as described previously[1]. The FibroScan Mini+ 430 (Echosens, Paris, France) was used with the M- or XL-probe according to body size and the suggestion by the device. LSM ≥7kPa was considered

suggestive for liver fibrosis[2]. CAP values ≥248 dB/m were considered suggestive of steatosis. CAP values were classified as low (<248 dB/m), intermediate (248-300 dB/m), and high (>300 dB/m). In addition, liver steatosis was semi-quantitatively assessed on ultrasound with an LogiQ S7 device with a C1-6-D probe (GE Healthcare, Diegem, Belgium) or the Butterfly IQ (Butterfly Network, New York, NY). Liver steatosis was classified as absent, mild and moderate/severe, based on liver-kidney contrast, liver echogenicity, and visibility of the intrahepatic vessels and diaphragm[3].

Blood samples were obtained after overnight fasting. Serum triglycerides, glucose, insulin and alanine aminotransferase (ALT) were determined. Serum fibroblast growth factor 21 (FGF21) and mitochondrial open reading frame of the 12S rRNA-c (MOTS-C) were determined using enzyme-linked immunosorbent assay (DF2100; R&D systems and ELK7627; ELK Biotechnology, respectively). Homeostatic model assessment for insulin resistance (HOMA-IR) was calculated as the product of fasting insulin (mIU/L) x glucose (mg/dL)/405.

Supplementary tables

Supplementary table 1. Genes and primers used for qPCR on murine liver samples.

Gene	Abbrevation	Forward primer	Reverse primer
Hydroxymethylbilane synthase	Hmbs	AAGGGCTTTTCTGAGGCACC	AGTTGCCCATCTTTCATCACTG
Hypoxanthine guanine phosphoribosyl transferase	Hprt	GTTAAGCAGTACAGCCCCAAA	AGGGCATATCCAACAACAAACTT
Glyceraldehyde 3 phosphate dehydrogenase	Gapdh	CATGGCCTTCCGTGTTCCTA	GCGGCACGTCAGATCCA
Sterol regulatory element binding protein 1	Srebp1c	TGACCCGGCTATTCCGTGA	CTGGGCTGAGCAATACAGTTC
Carnitine palmitoyltransferase 1a	Cpt1a	CTCCGCCTGAGCCATGAAG	CACCAGTGATGATGCCATTCT
Tumor necrosis factor alpha	Tnfalpha	CATCTTCTCAAAATTCGAGTGACAA	TGGGAGTAGACAAGGTACAACCC
Collagen type I alpha 1	Col1a1	GCTCCTCTTAGGGGCCACT	CCACGTCTCACCATTGGGG
NADH dehydrogenase 1	Nd1	TGCACCTACCCTATCACTC	ATTGTTTGGGCTACGGCTC
Cytochrome b	Cytb	TACCTGCCCCATCCAACATT	TAAGCCTCGTCCGACATGAA
Cytochrome c oxidase I	Co1	ACCCAGATGCTTACACCACA	TGTGATATGGTGGAGGGCAG
ATP synthase 6	Atp6	CCACACACCAAAAGGACGAA	GAAGGAAGTGGGCAAGTGAG
peroxisome proliferative activated receptor gamma coactivator 1 alpha	Pgc1alpha	TCTCAGTAAGGGGCTGGTTG	TGACGCCAGTCAAGCTTTTTC
Nuclear respiratory factor 1	Nrf1	TATGGCGGAAGTAATGAAAGACG	CAACGTAAGCTCTGCCTTGTT
Transcription factor A	Tfam	GGAATGTGGAGCGTGCTAAAA	ACAAGACTGATAGACGAGGGG
OPA1 mitochondrial dynamin like GTPase	Opa1	TGGAAAATGGTTCGAGAGTCAG	CATTCCGTCTCTAGGTTAAAGCG
Mitofusin 1	Mfn1	CCTACTGCTCCTTCTAACCCA	AGGGACGCCAATCCTGTGA
Dynamin 1-like	Dnm1l	TTACGGTTCCCTAAACTTCACG	GTCACGGGCAACCTTTTACGA
Dynamin 2	Dnm2	TTTGGCGTTCGAGGCCATT	CAGGTCCACGCATTTCAGAC
Fission, mitochondrial 1	Fis1	AGGCTCTAAAGTATGTGCGAGG	GGCCTTATCAATCAGGCGTTC
BCL-2 interacting protein 3	Bnip3	TCCTGGGTAGAACTGCACTTC	GCTGGGCATCCAACAGTATTT
Parkin	Parkin	GAGGTCCAGCAGTTAAACCCA	CACACTGAACTCGGAGCTTTC

Supplementary table 2. Genes and primers used for determination of mitochondrial DNA copy number by RT-qPCR.

Gene	Forward primer	Reverse primer
D-loop	AGGCATGAAAGGACAGCA	TTGGCATTAAGAGGAGGG
18S rRNA	GAGAAACGGCTACCACATCC	CACCAGACTTGCCCTCCA

Supplementary figures

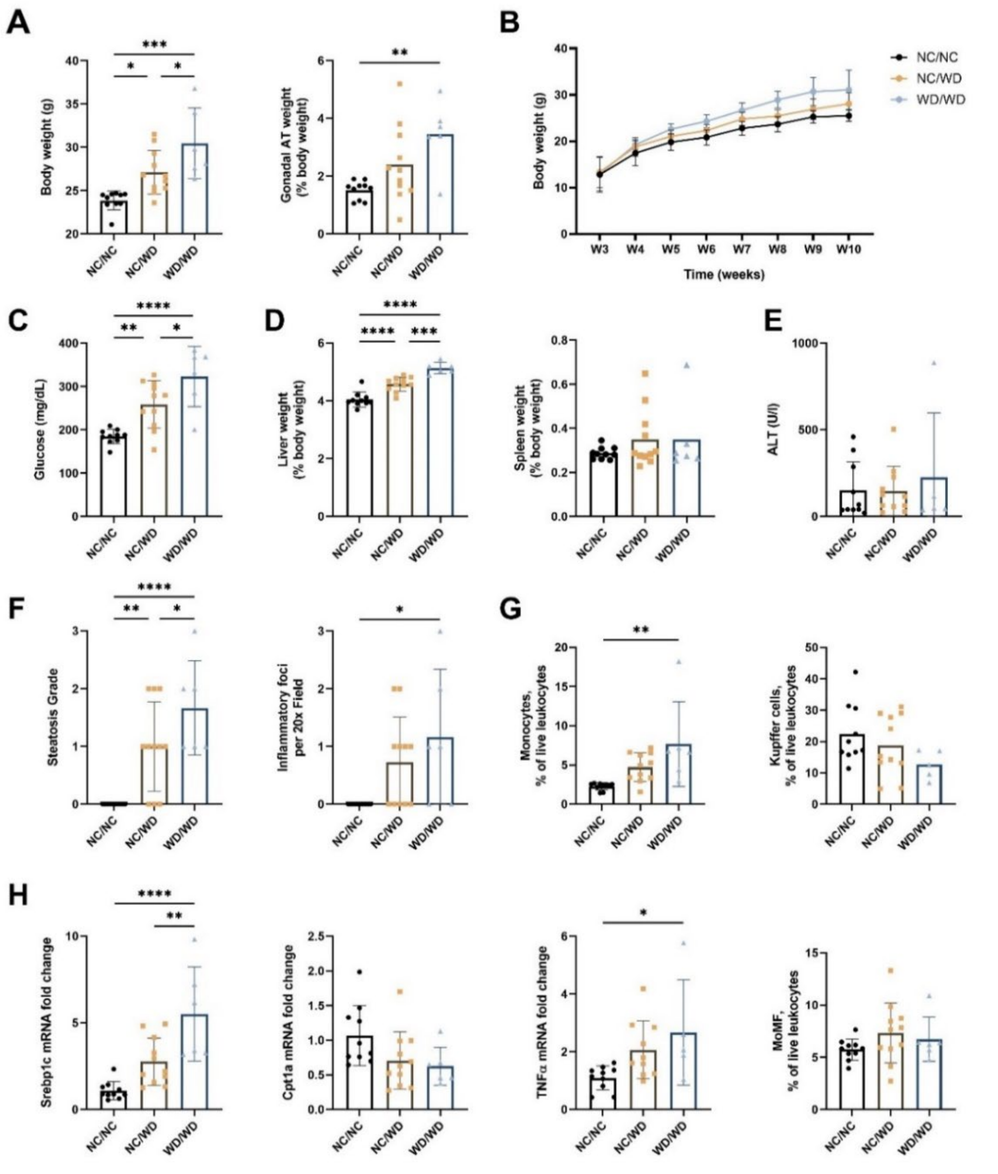

Fig. S1. Effect of maternal WD on 10-week-old male offspring. Body weight and relative gonadal adipose tissue weight (A). Body weight evolution (B). Serum glucose level (C). Relative liver and spleen weight (D). Serum ALT levels (E). Scoring of steatosis grade and inflammatory cell infiltration (F). Relative cell quantification of monocytes, KCs and MoMFs (G). Relative gene expression of *Srebp1c*, *Cpt1a* and *Tnfα* (H). Data are presented as mean ± SD. Statistical significance was evaluated by one-way ANOVA followed by Tukey post-hoc testing. * P<0.05; ** P<0.01; *** P<0.001; **** P<0.0001.

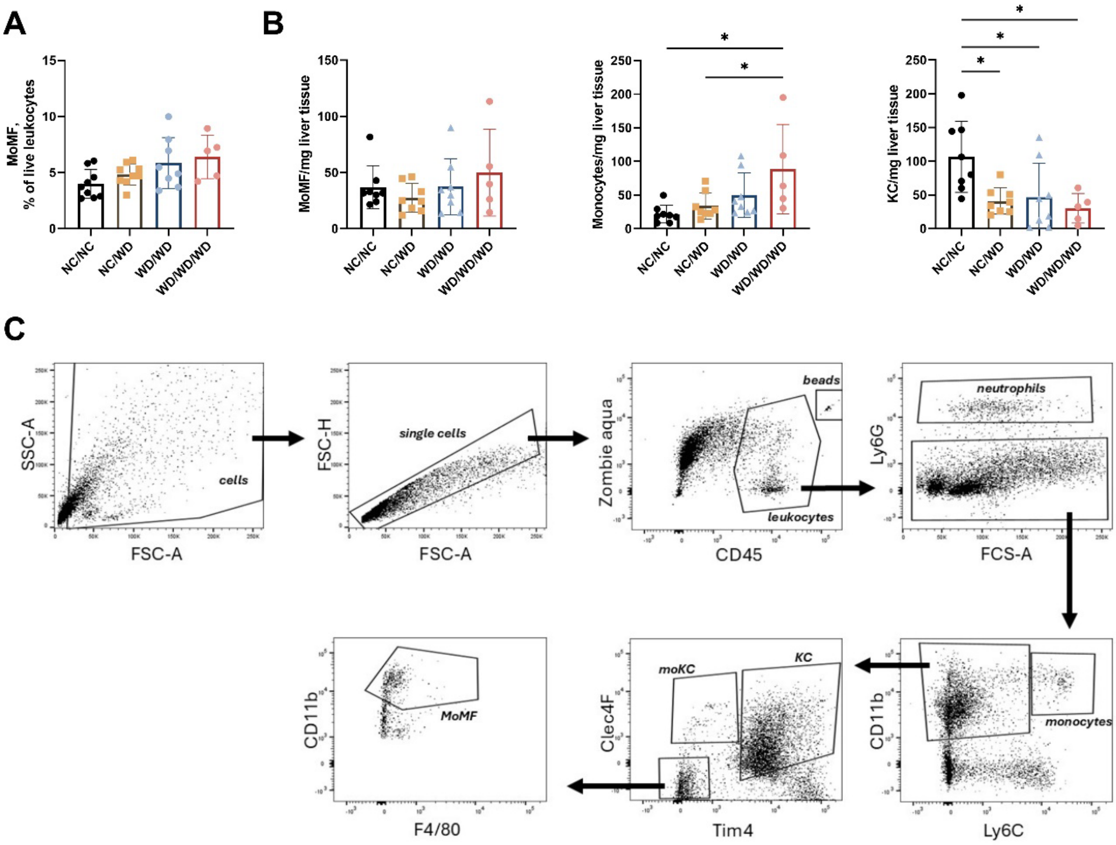

Fig. S2, related to figure 2. Effect of (multigenerational) maternal WD on MASLD development in male offspring at 16 and 3 weeks of age. Relative cell quantification of monocyte-derived macrophages (MoMF) (A). Absolute cell quantification per liver tissue weight of MoMF, monocytes and KCs (B). Gating strategy for flow cytometry analysis of the liver (C). Data are presented as mean ± SD. Statistical significance was evaluated by one-way ANOVA followed by Tukey post-hoc testing. * P<0.05.

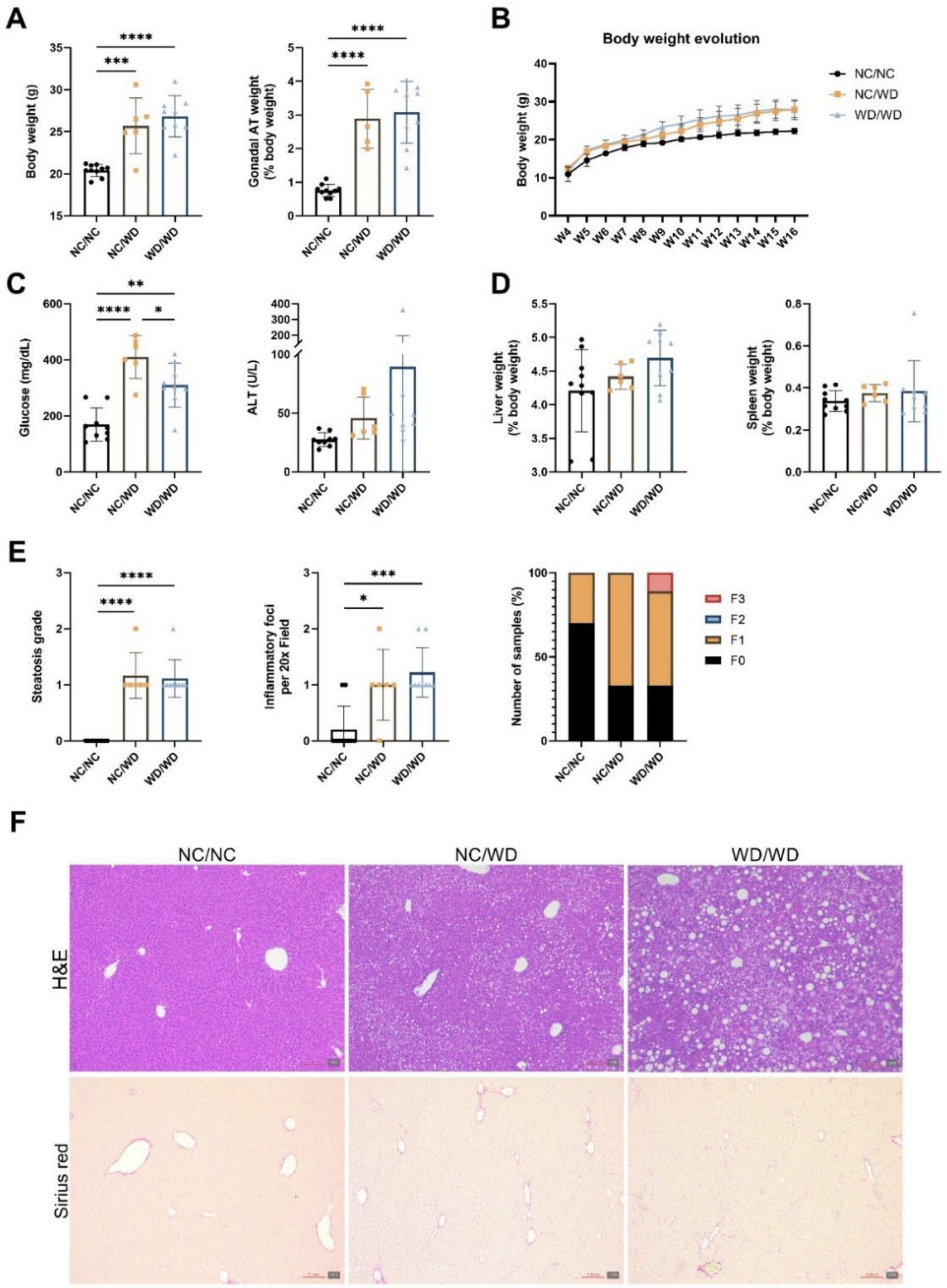

Fig. S3. Effect of maternal WD on female 16-week-old offspring. Body weight and relative gonadal adipose tissue weight (A). Body weight evolution (B). Serum glucose and ALT levels (C). Relative liver and spleen weight (D). Scoring of steatosis grade, inflammatory cell infiltration and fibrosis (E). Representative images of H&E and Sirius red stained slides (scale bar = 100μm) (F). Data are presented as mean ± SD. Statistical significance was evaluated by one-way ANOVA followed by Tukey post-hoc testing. * P<0.05; ** P<0.01; *** P<0.001; **** P<0.0001.

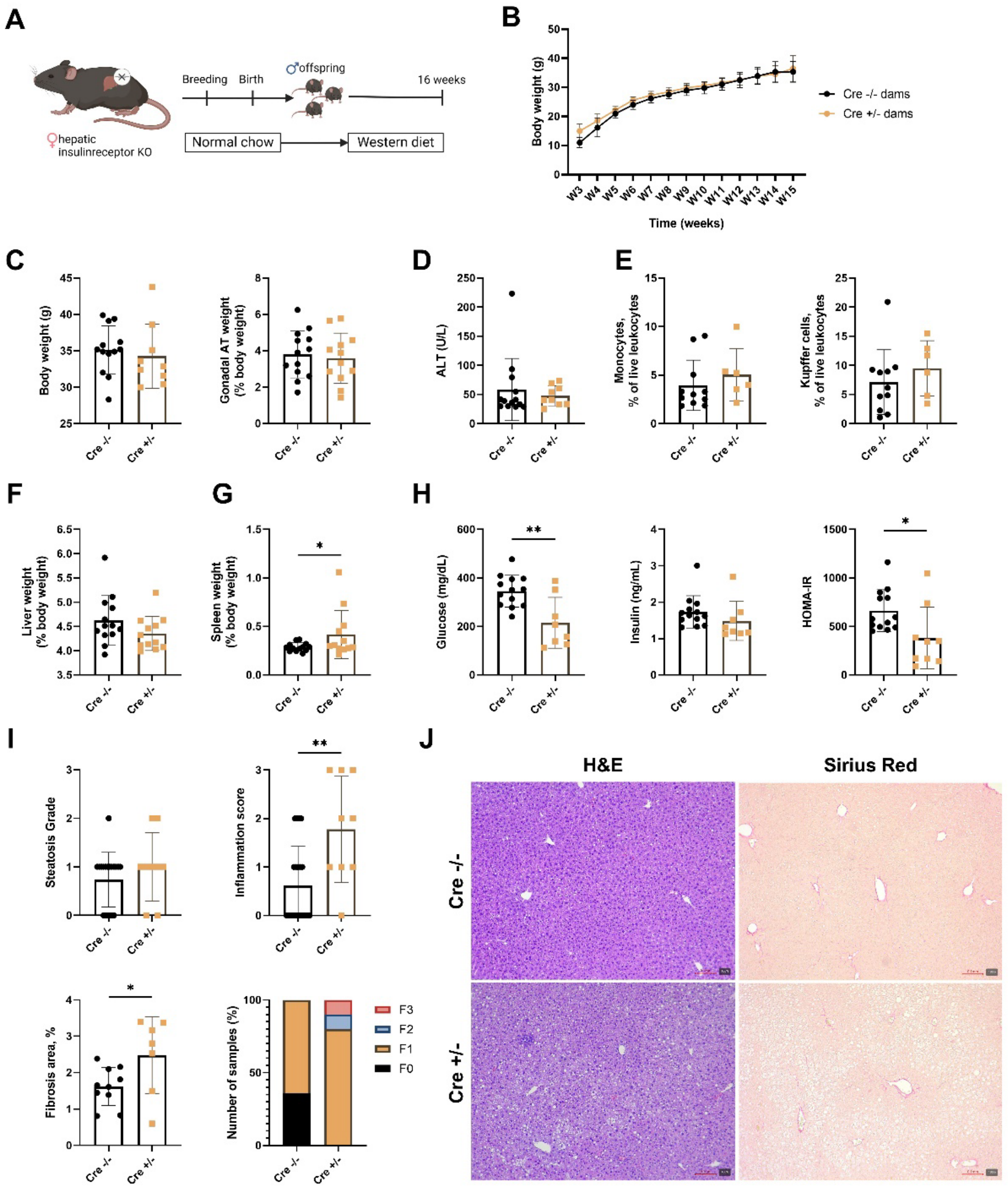

Fig. S4. Effect of maternal hepatic insulin resistance on male offspring. Schematic overview of the model. Created with BioRender (A). Body weight evolution (B), body and gonadal AT weight (C) and serum ALT levels (D) of 16-week-old offspring. Relative cell quantification of monocytes and KCs (E). Relative liver (F) and spleen weight (G). Serum glucose and insulin levels and HOMA-IR (H). Scoring of steatosis grade, inflammation and fibrosis and quantification of Sirius red area (I). Representative images of H&E and Sirius red stained slides (scale bars = 100µm) (J). Data are presented as mean ± SD. Statistical significance was evaluated by the unpaired student t-test. * P<0.05; ** P<0.01.

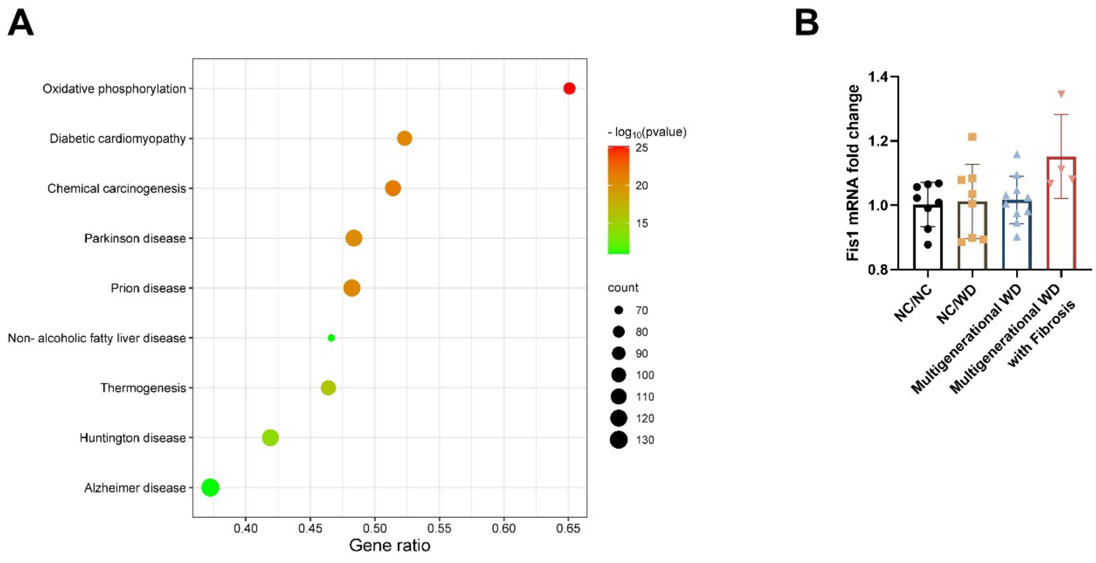

Fig. S5, related to figure 3. Effect of maternal WD on liver transcriptomics of 16-week-old offspring. Differentially regulated pathways between NC/WD and multigenerational WD + fibrosis after KEGG pathway analysis, plotted with p-value (shading), number of differentially regulated genes (circle size) and percentage of differentially regulated genes (x-axis) (A). Relative gene expression of *Fis1* (mitochondrial fission) (B). Data are presented as mean ± SD. Statistical significance was evaluated by one-way ANOVA followed by Tukey post-hoc testing.

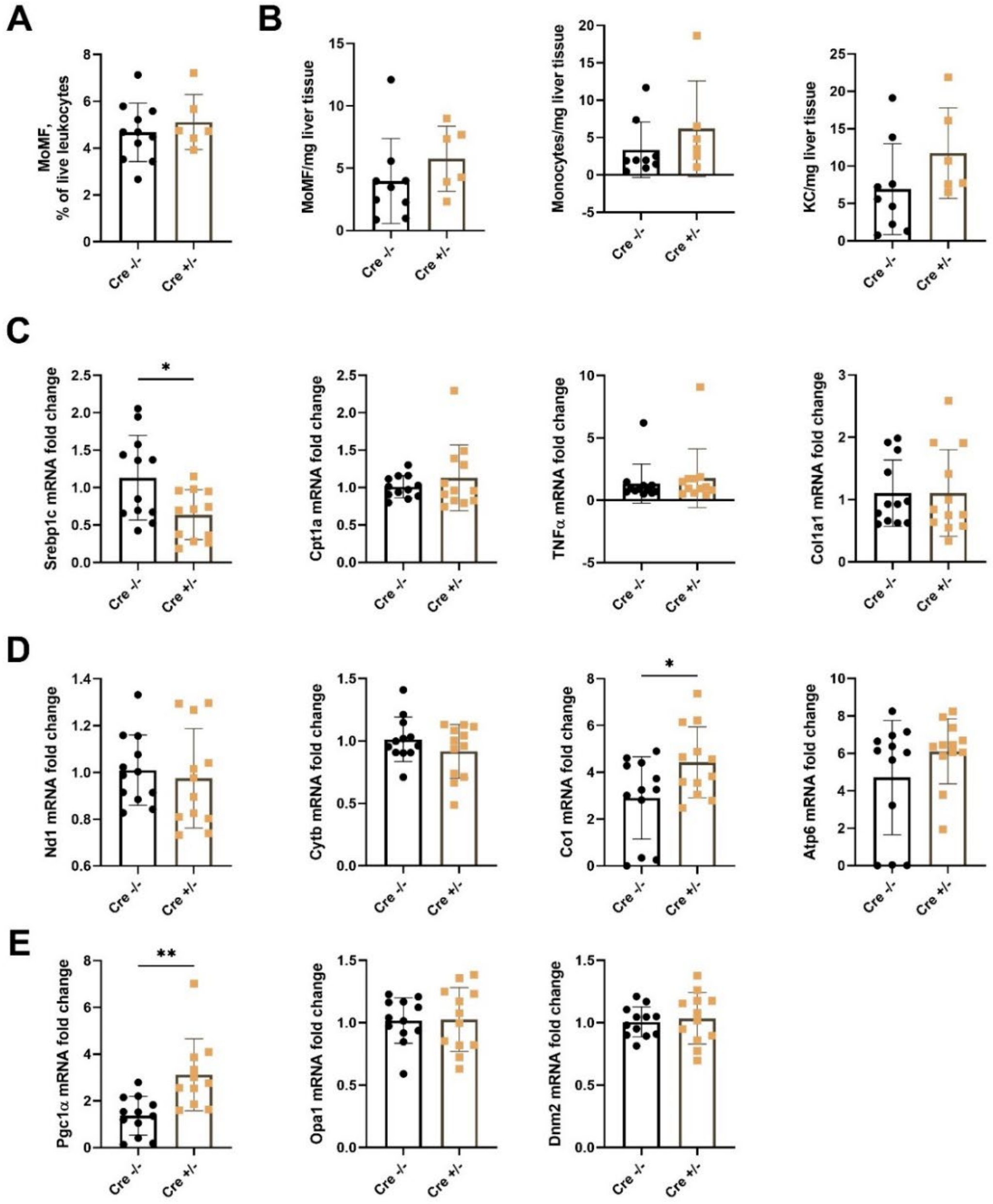

Fig. S6, related to Fig. S4. Effect of maternal hepatic insulin resistance on male offspring. Relative cell quantification of MoMFs (A). Absolute cell quantification per liver tissue weight of MoMFs, monocytes and KCs (B). Relative gene expression of *Srebp1c*, *Cpt1a*, *Tnfα* and *Col1a1* (C). Relative gene expression levels of mitochondrial-encoded OXPHOS subunits (*Nd1*, *Cytb*, *Co1*, and *Atp6*) (D). Relative gene expression levels of *Pgc1alpha*, *Opa1*, and *Dnm2* (E). Data are presented as mean ± SD. Statistical significance was evaluated by the unpaired student t-test. * P<0.05; ** P<0.01; *** P<0.001; **** P<0.0001.

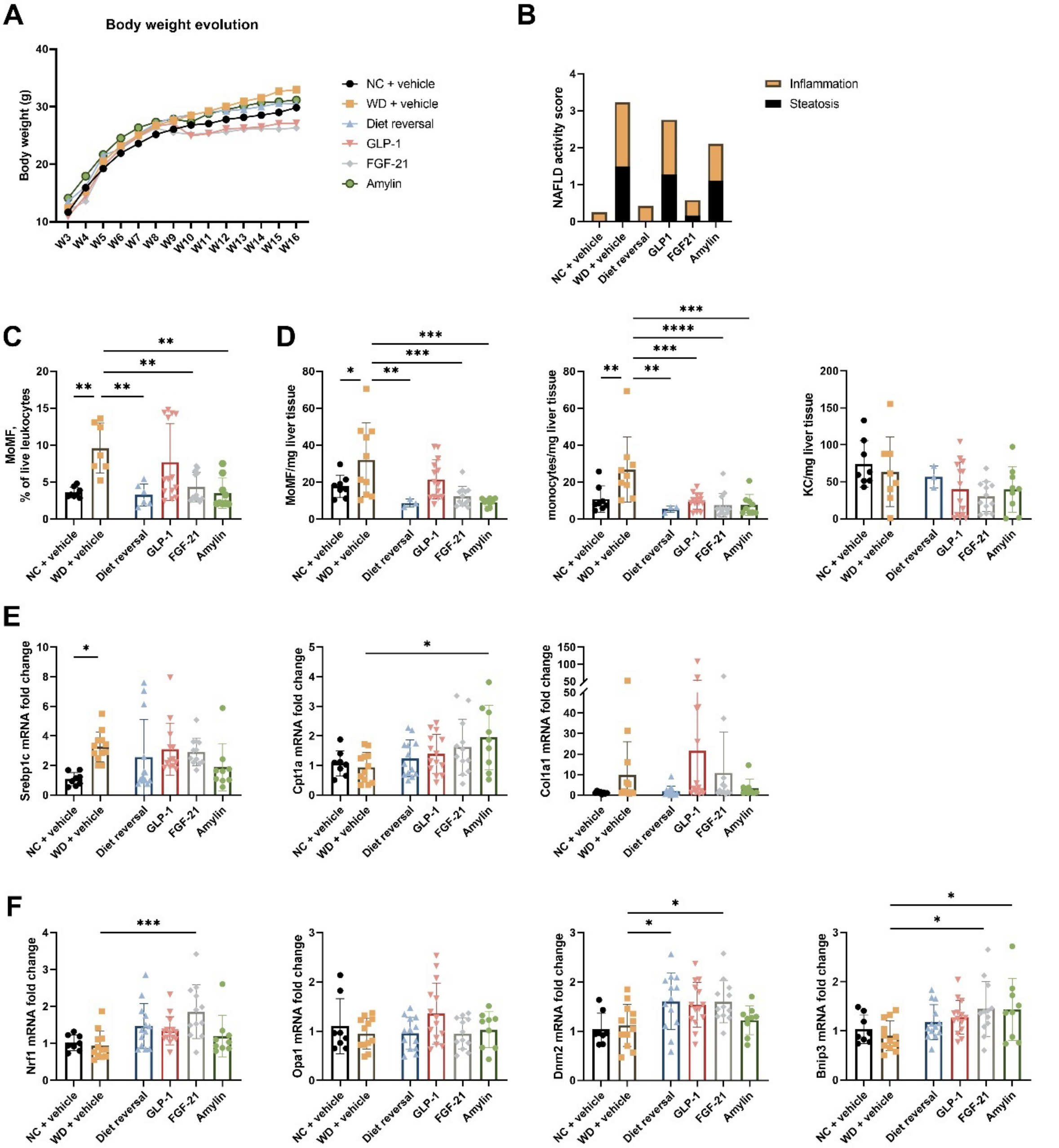

Fig. S7 related to figure 6. Evaluation of pharmacological compounds in the maternal WD model. Body weight evolution (A). MASLD activity score (B). Relative cell quantification of MoMFs (C). Absolute cell quantification per liver tissue weight of MoMFs, monocytes and KCs (D). Relative gene expression of *Srebp1c*, *Cpt1a*, *Tnfα* and *Col1a1* (E). Relative gene expression of *Nrf1*, *Opa1*, *Dnm2,* and *Bnip3* (F). Data are presented as mean ± SD. Statistical significance was evaluated by one-way ANOVA followed by Tukey post-hoc testing. * P<0.05; ** P<0.01; *** P<0.001; **** P<0.0001.

Supplementary references

[1] Lefere S, Dupont E, De Guchtenaere A, Van Biervliet S, Vande Velde S, Verhelst X, et al. Intensive Lifestyle Management Improves Steatosis and Fibrosis in Pediatric Nonalcoholic Fatty Liver Disease. Clinical Gastroenterology and Hepatology 2022;20:2317-2326.e4. https://doi.org/10.1016/j.cgh.2021.11.039.

[2] Nobili V, Vizzutti F, Arena U, Abraldes JG, Marra F, Pietrobattista A, et al. Accuracy and reproducibility of transient elastography for the diagnosis of fibrosis in pediatric nonalcoholic steatohepatitis. Hepatology 2008;48:442–8. https://doi.org/10.1002/hep.22376.

[3] Shannon A, Alkhouri N, Carter-Kent C, Monti L, Devito R, Lopez R, et al. Ultrasonographic quantitative estimation of hepatic steatosis in children With NAFLD. J Pediatr Gastroenterol Nutr 2011;53:190–5. https://doi.org/10.1097/MPG.0b013e31821b4b61.
