# Supplementary material for: Mitochondrial dysfunction characterises the multigenerational effects of maternal obesity on MASLD
Source: JHEP Rep. 2025 Mar 29;7(6):101404. doi: 10.1016/j.jhepr.2025.101404 (PMC12151218; doi:10.1016/j.jhepr.2025.101404)
Supplement: Multimedia component 2 [file mmc2.docx]

**JHEP Reports**

**CTAT methods**

Tables for a “Complete, Transparent, Accurate and Timely account” (CTAT) are now mandatory for all revised submissions. The aim is to enhance the reproducibility of methods.

- Only include the parts relevant to your study
- Refer to the CTAT in the main text as ‘Supplementary CTAT Table’
- Do not add subheadings
- Add as many rows as needed to include all information
- Only include one item per row

**If the CTAT form is not relevant to your study, please outline the reasons why:**

|  |
| --- |

- 1. **Antibodies**

| **Name** | **Citation** | **Supplier** | **Cat no.** | **Clone no.** |
| --- | --- | --- | --- | --- |
|  |  |  |  |  |

- 1. **Cell lines**

| **Name** | **Citation** | **Supplier** | **Cat no.** | **Passage no.** | **Authentication test method** |
| --- | --- | --- | --- | --- | --- |
|  |  |  |  |  |  |

- 1. **Organisms**

| **Name** | **Citation** | **Supplier** | **Strain** | **Sex** | **Age** | **Overall n number** |
| --- | --- | --- | --- | --- | --- | --- |
| **Mice** |  | **Janvier** | **C57BL/6** | **Male and female** | **3-16 weeks** | **128** |
| **Mice** |  | **The Jackson Laboratory** | **Alb-CreIR^fl/fl^** | **Male** | **16 weeks** | **21** |

- 1. **Sequence based reagents**

| **Name** | **Sequence** | **Supplier** |
| --- | --- | --- |
| **Hmbs** | AAGGGCTTTTCTGAGGCACC AGTTGCCCATCTTTCATCACTG | **Bioline** |
| **Hprt** | GTTAAGCAGTACAGCCCCAAA AGGGCATATCCAACAACAAACTT | **Bioline** |
| **Gapdh** | CATGGCCTTCCGTGTTCCTA GCGGCACGTCAGATCCA | **Bioline** |
| **Srebp1c** | TGACCCGGCTATTCCGTGA  CTGGGCTGAGCAATACAGTTC | **Bioline** |
| **Cpt1a** | CTCCGCCTGAGCCATGAAG  CACCAGTGATGATGCCATTCT | **Bioline** |
| **Tnfalpha** | CATCTTCTCAAAATTCGAGTGACAA TGGGAGTAGACAAGGTACAACCC | **Bioline** |
| **Col1a1** | GCTCCTCTTAGGGGCCACT  CCACGTCTCACCATTGGGG | **Bioline** |
| **Nd1** | TGCACCTACCCTATCACTC ATTGTTTGGGCTACGGCTC | **Bioline** |
| **Cytb** | TACCTGCCCCATCCAACATT TAAGCCTCGTCCGACATGAA | **Bioline** |
| **Co1** | ACCCAGATGCTTACACCACA TGTGATATGGTGGAGGGCAG | **Bioline** |
| **Atp6** | CCACACACCAAAAGGACGAA GAAGGAAGTGGGCAAGTGAG | **Bioline** |
| **Pgc1alpha** | TCTCAGTAAGGGGCTGGTTG  TGACGCCAGTCAAGCTTTTTC | **Bioline** |
| **Nrf1** | TATGGCGGAAGTAATGAAAGACG  CAACGTAAGCTCTGCCTTGTT | **Bioline** |
| **Tfam** | GGAATGTGGAGCGTGCTAAAA  ACAAGACTGATAGACGAGGGG | **Bioline** |
| **Opa1** | TGGAAAATGGTTCGAGAGTCAG  CATTCCGTCTCTAGGTTAAAGCG | **Bioline** |
| **Mfn1** | CCTACTGCTCCTTCTAACCCA AGGGACGCCAATCCTGTGA | **Bioline** |
| **Dnm1l** | TTACGGTTCCCTAAACTTCACG  GTCACGGGCAACCTTTTACGA | **Bioline** |
| **Dnm2** | TTTGGCGTTCGAGGCCATT  CAGGTCCACGCATTTCAGAC | **Bioline** |
| **Fis1** | AGGCTCTAAAGTATGTGCGAGG  GGCCTTATCAATCAGGCGTTC | **Bioline** |
| **Bnip3** | TCCTGGGTAGAACTGCACTTC GCTGGGCATCCAACAGTATTT | **Bioline** |
| **Parkin** | GAGGTCCAGCAGTTAAACCCA GCTGGGCATCCAACAGTATTT | **Bioline** |
| **D-loop** | AGGCATGAAAGGACAGCA TTGGCATTAAGAGGAGGG | **Bioline** |
| **18S rRNA** | GAGAAACGGCTACCACATCC CACCAGACTTGCCCTCCA | **Bioline** |

- 1. **Biological samples**

| **Description** | **Source** | **Identifier** |
| --- | --- | --- |
| Human serum samples | **Zeepreventorium** |  |

- 1. **Deposited data**

| **Name of repository** | **Identifier** | **Link** |
| --- | --- | --- |
| NCBI | GSE291363 |  |

- 1. **Software**

| **Software name** | **Manufacturer** | **Version** |
| --- | --- | --- |
| **SPSS** | **IBM Corp** | **27.0** |
| **Graphpad Prism** | **Graphpad Prism** | **8** |
| **R** | **The R foundation** | **4.2.1.** |

- 1. **Other (*e.g*. drugs, proteins, vectors etc.)**

| FGF21 NNC0194-0001 | Novo Nordisk |  |
| --- | --- | --- |
| Semaglutide | Novo Nordisk |  |
| Amylin analogue NNC0174-0839 | Novo Nordisk |  |

- 1. **Please provide the details of the corresponding methods author for the manuscript:**

| Sander Lefere  The Core, Corneel Heymanslaan 10, 9000 Gent  Tel.: +32 9 332 2361  sander.lefere@ugent.be |
| --- |

**2.0 Please confirm for randomised controlled trials all versions of the clinical protocol are included in the submission. These will be published online as supplementary information.**

| N/A |
| --- |
