# Supplementary material for: Mitochondrial dysfunction characterises the multigenerational effects of maternal obesity on MASLD
Source: JHEP Rep. 2025 Mar 29;7(6):101404. doi: 10.1016/j.jhepr.2025.101404 (PMC12151218; doi:10.1016/j.jhepr.2025.101404)
Supplement: Multimedia component 4 [file mmc4.pdf]

Mitochondrial dysfunction characterises the multigenerational effects of maternal obesity on MASLD

Anneleen Heldens[1,2], Milton Antwi[1,2,3,4], Louis Onghena[1,2,5], Tim Meese[6], Yannick Gansemans[6], Joél Smet[7], Ellen Dupont[8], Xavier Verhelst[1,2], Sarah Raevens[1,2], Hans Van Vlierberghe[1,2], Arnaud Vanlander[7], Filip Van Nieuwerburgh[6], Lindsey Devisscher[1,3], Ruth De Bruyne[9], Anja Geerts[1,2], Sander Lefere[1,2,*]

JHEP Reports **2025**. vol. 7 | 1–13

Check for updates

Background & Aims: Although maternal obesity is an independent risk factor for metabolic dysfunction-associated steatotic liver disease (MASLD), the pathogenesis remains unclear. We aimed to evaluate the effect and mechanisms of multigenerational maternal Western diet (WD) on MASLD progression, and test drug candidates.

Methods: Female mice were fed WD from 8 weeks before breeding initiation with a normal chow (NC)-fed male, throughout pregnancy and lactation. Male offspring were weaned onto NC or WD and assessed at the age of 24 days, 10 weeks, and 16 weeks (n = 5–11 per group). Additionally, offspring from dams with hepatic insulin receptor knockout were evaluated (n = 9–12 per group). Serum fibroblast growth factor 21 (FGF21) and mitochondrial open reading frame of 12S rRNA-c (MOTS-c) were measured in adolescents with MASLD with or without a history of maternal obesity. The therapeutic efficacy of FGF21, semaglutide and an amylin analogue was assessed from 8 to 16 weeks of age (n = 8–12 per group).

Results: Starting from weaning age, maternal WD feeding aggravated body weight gain, insulin resistance, steatosis, and inflammation. Fibrosis was only observed in offspring exposed to maternal WD. Mechanistically, the latter exhibited reduced OXPHOS activity. Isolated maternal hepatic insulin resistance partially recapitulated offspring inflammation and fibrosis. Notably, OXPHOS was also downregulated in a transcriptomic dataset of maternal WD feeding in non-human primates. Serum FGF21 and MOTS-c correlated with MASLD severity and maternal obesity in adolescents. Particularly FGF21 treatment ameliorated steatohepatitis and mitochondrial function.

Conclusions: Maternal WD aggravates MASLD in male offspring starting from weaning age, with mitochondrial dysfunction contributing to disease severity. This was reversed by FGF21 agonism.

© 2025 The Author(s). Published by Elsevier B.V. on behalf of European Association for the Study of the Liver (EASL). This is an open access article under the CC BY license (http://creativecommons.org/licenses/by/4.0/).

Introduction

Metabolic dysfunction-associated steatotic liver disease (MASLD) has become the most common chronic liver disease in both children and adults, with an estimated prevalence of 7.6% and 30%, respectively.[1,2] MASLD encompasses a wide spectrum from simple steatosis to hepatic inflammation (metabolic-associated steatohepatitis; MASH) with or without fibrosis. Despite not fully understanding the underlying mechanisms, maternal obesity has been identified as a major risk factor of MASLD and obesity in adolescents, which is associated with the development of severe liver disease later in life.[3–5] In addition, about 20% of European women of childbearing age have obesity, emphasising the importance of studying the effects of maternal obesity on offspring health.[6,7]

One point of contention is the nature of the deleterious maternal factors. Although most preclinical studies rely on a Western-style diet inducing variable degrees of insulin resistance (IR) or obesity, it has recently been suggested that isolated (genetic) maternal IR alone is sufficient to drive offspring MASLD.[8] Furthermore, preclinical studies have linked maternal obesity to offspring hepatic mitochondrial dysfunction, including impaired oxidative phosphorylation (OXPHOS) capacity and oxidative stress.[9–11] Importantly, mitochondrial dysfunction is associated with MASH progression in patients, with evidence for structural changes of the mitochondria, OXPHOS capacity reduction, increased reactive oxygen species and oxidative DNA damage.[12,13] Mitochondria, and their embedded mitochondrial DNA are exclusively maternally inherited. Therefore, these organelles might also mediate the potential effect of maternal obesity over several generations,[14,15] although this remains unclear.

Although multiple preclinical studies have investigated the metabolic effect of maternal obesity, MASLD was often not studied in depth, and pharmacological interventions have not been tested. Currently, several compounds are in clinical trial for MASLD. Semaglutide is a glucagon-like peptide 1 (GLP-1) analogue, which improves MASLD indirectly by ameliorating

* Corresponding author. Address: Hepatology Research Unit, Dpt. Internal Medicine and Pediatrics; Ghent University, The Core, Entrance 37, Corneel Heymanslaan 10, B-9000 Ghent, Belgium; Tel. +32 9 332 2361 (S. Lefere).
E-mail address: sander.lefere@ugent.be (S. Lefere).
https://doi.org/10.1016/j.jhepr.2025.101404

obesity, as there is no hepatic expression of GLP-1 receptors.[16] In a phase II clinical trial, semaglutide improved steatohepatitis, but not fibrosis.[17] A second potential therapeutic strategy is fibroblast growth factor 21 (FGF21), a hormone produced mainly by the liver, with pleiotropic metabolic actions both in the liver and systemically, including stimulation of mitochondrial biogenesis and fatty acid oxidation (FAO). In animal models, FGF21 administration improved MASH by reducing oxidative stress and enhancing mitochondrial function.[18] Recently, FGF21 analogues have been shown to improve MASH and fibrosis in clinical trials phase IIb.[19,20] Amylin analogues induce weight loss through central hypothalamic effects.[21] Evaluation is limited to preclinical research, but dual calcitonin–amylin agonists have shown improvement of steatosis.[22]

We aimed to explore the consequences and underlying mechanisms of maternal obesity on MASLD development in the offspring, revealing reduction in OXPHOS function as a key contributor. These findings were translated to non-human primates and adolescents with obesity. Furthermore, potential accumulating effects of multigenerational exposure to maternal obesity were evaluated. Finally, several potential compounds for MASLD treatment, namely semaglutide, FGF21 and an amylin analogue, were assessed in our model.

Materials and methods

Part of the methods are described in the supplementary file.

Animals

Mice were housed in open cages at the animal facility of the Ghent University Hospital in a 12-h light/dark cycle at 21–23 °C with free access to food and water. All *in vivo* experiments were approved by the Animal Ethics Committee of the Ghent University's Faculty of Medicine and Health Sciences (Approval 20/04 and 22/63).

Diet-induced model of maternal obesity

Eight-week-old female C57BL/6J mice (Janvier Labs, Le Genest-Saint-Isle, France) were fed either Western diet (WD) (TD.08811 + 1% cholesterol; Sniff, Soest, Germany), which is rich in saturated fat, sucrose and cholesterol, or normal chow (NC) control for 8 weeks before breeding to a NC-fed male. Diet was maintained during breeding and lactation. Female offspring were weaned onto NC and used to produce the next generation in the same way as the first generation. Offspring from the second generation were weaned onto WD or NC. In this way, four groups of offspring were generated: NC-fed offspring without maternal obesity (healthy controls, NC/NC), WD-fed offspring without maternal obesity (NC/WD), and WD-fed offspring with one or two generations of maternal obesity (WD/WD and WD/WD/WD respectively). Metabolic alterations and MASLD development in offspring were evaluated at weaning age (24 days), 10 weeks, and 16 weeks of age.

Therapeutic study of metabolic compounds

The above-described mouse model was used for pharmacological studies. At 8 weeks of age, WD/WD mice were randomised to the diet reversal group, by switching to NC feeding, or to a treatment group. The latter received either vehicle (50 mM sodium phosphate + 70 mM sodium chloride), the GLP-1 analogue semaglutide (30 nmol/kg once daily), wild-type FGF21 (0.3 mg/kg twice daily) or an amylin analogue (NN0174-0839; 10 nmol/kg once daily) via subcutaneous injection for 8 weeks. Up-titration of semaglutide was performed over 6 days. WD feeding during treatment was maintained. Amylin (NNC0174-0839) was provided by Novo Nordisk Compound Sharing, and wild-type FGF21 (NNC0194-0001) as well as semaglutide were provided by Novo Nordisk A/S, Måløv, Denmark.

Genetic model of maternal hepatic insulin resistance

Female homozygous insulin receptor-floxed ($IR^{lox/lox}$) mice and transgenic male mice expressing Cre recombinase under the control of the hepatocyte-specific albumin promotor (Alb-Cre) were purchased from The Jackson Laboratory. $IR^{lox/lox}$ and Alb-Cre mice were crossed to generate liver-specific insulin receptor knockout (LIRKO) mice. Female LIRKO and $IR^{lox/lox}$ NC-fed mice were both bred to an $IR^{lox/lox}$ male mouse. Only Cre-negative male offspring (from LIRKO and control parents) were used in the experiment and sacrificed at 16 weeks of age.

Statistical analysis

Statistical analysis was performed and graphical representations were made using SPSS 27.0 (SPSS Software, IBM Corp, Armonk, NY), R (version 4.2.1), and GraphPad Prism 8 (GraphPad Software Inc., USA). Variables were tested for normality using Shapiro-Wilk normality test. A two-sided p value <0.05 was considered statistically significant. For preclinical data, continuous variables are presented as mean ± SD. Multiple group comparisons were performed using a one-way ANOVA with post-hoc testing. An unpaired Student t test was performed to compare two groups. For clinical data, normally distributed data are presented as mean ± SD, otherwise data are presented as median ± IQR. Where appropriate, statistical significance is evaluated using the Kruskal–Wallis test followed by Dunn's *post-hoc* testing, Mann–Whitney test or unpaired Student t test. Correlations between continuous variables were assessed using Spearman's rank correlation test.

Results

Multigenerational maternal WD feeding worsens male offspring metabolic health starting from weaning age

Female C57BL/6J mice were either fed a NC control diet or WD before breeding and during pregnancy and lactation, with offspring mice weaned to either of these diets (NC/NC: n = 8; NC/WD: n = 9; WD/WD: n = 9; WD/WD/WD: n = 5) (Fig. 1A). In 16-week-old male offspring, postnatal WD significantly increased body weight and gonadal adipose tissue (AT) weight, which can be considered a measure for central obesity. This was exacerbated in a stepwise fashion with increasing exposure to maternal WD feeding (Fig. 1B and C). Glucose tolerance, estimated by intraperitoneal glucose tolerance test (IPGTT), was mainly determined by the postnatal diet (Fig. 1D). Furthermore, WD/WD mice were more insulin resistant, based on the homeostatic model assessment of insulin resistance (HOMA-IR), compared to NC/WD mice, which was further aggravated by

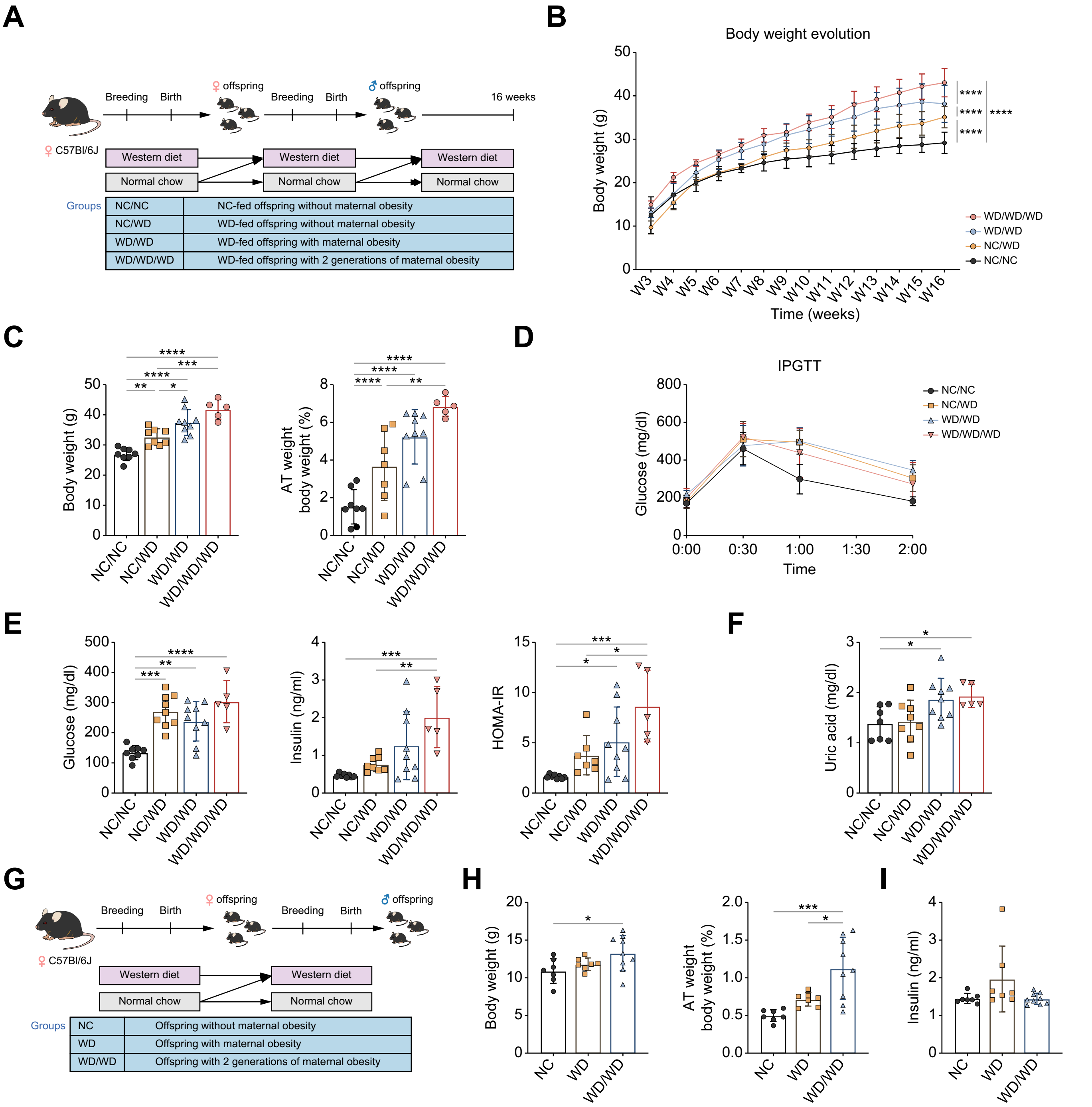

Fig. 1. Effect of (multigenerational) maternal WD on metabolic characteristics of male offspring at 16 and 3 weeks of age. Schematic overview of the maternal WD model to evaluate the 16-week-old offspring. Created with BioRender (A). Body weight evolution (B), body and relative gonadal AT weight (C), and IPGTT of the 16-week-old offspring (D). Serum glucose and insulin levels and HOMA-IR (E) and serum uric acid levels (F). Schematic overview of the maternal WD model to evaluate 3-week-old offspring. Created with BioRender (G). Body and relative gonadal AT weight (H) and serum insulin levels (I) of the 3-week-old offspring. Data are presented as mean ± SD. Statistical significance was evaluated by one-way ANOVA followed by Tukey *post-hoc* testing. **p* <0.05; ***p* <0.01; ****p* <0.001; *****p* <0.0001. AT, adipose tissue; HOMA-IR, homeostatic model assessment of insulin resistance; IPGTT, intraperitoneal glucose tolerance test; NC, normal chow; WD, Western diet.

multigenerational maternal WD feeding (Fig.1E). Serum uric acid levels, which is associated with hepatic steatosis in patients, were only elevated in offspring exposed to maternal WD feeding (Fig. 1F). Similar to the offspring at 16 weeks of age, body and gonadal AT weight and serum glucose levels were increased in 10-week-old WD/WD mice (Fig. S1A–C).

We further investigated the impact of isolated maternal WD feeding on the offspring's health. Therefore, to exclude the effect of postnatal diet on MASLD development, the offspring were sacrificed at weaning age (24 days). Surprisingly, multigenerational maternal WD feeding (n = 9) significantly increased the offspring body and gonadal AT weight, without affecting

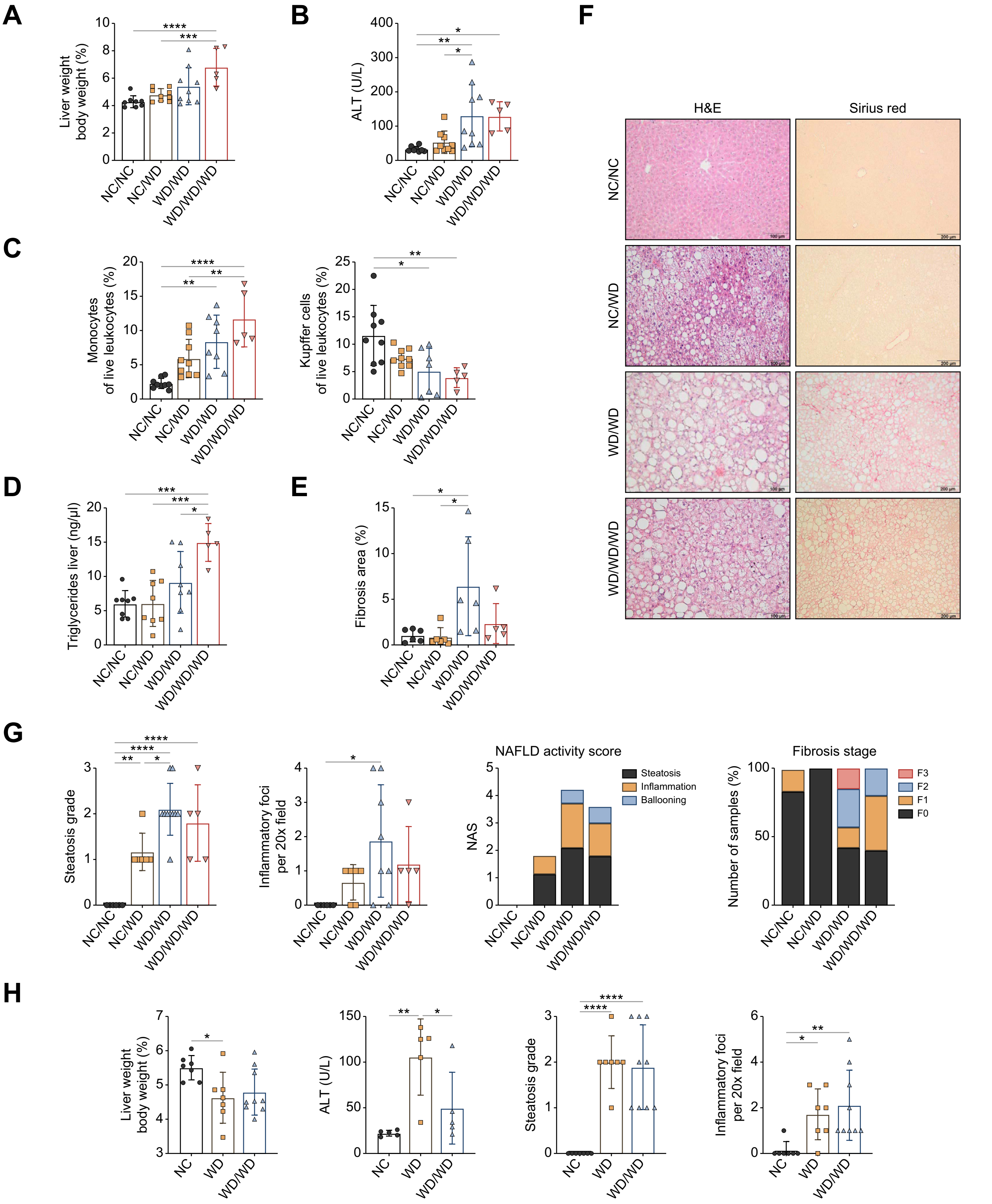

Fig. 2. Effect of (multigenerational) maternal WD on MASLD development in male offspring at 16 and 3 weeks of age. Relative liver weight (A) and serum ALT levels (B) of the 16-week-old offspring. Relative cell quantification of hepatic monocytes and KCs (C). Quantification of triglyceride content (D) and Sirius Red area (E). Representative images of H&E and Sirius Red stained slides (scale bars = 100 µm and 200 µm, respectively) (F), with scoring of steatosis grade, inflammatory cell infiltration, NAFLD activity sore and fibrosis stage of the 16-week-old offspring (G). Relative liver weight, serum ALT levels, and scoring of steatosis grade and

serum insulin levels, compared with healthy control mice (n = 7) and exposure to one generation of maternal WD (n = 7) (Fig. 1H and I).

Diet-induced MASLD is aggravated by multigenerational maternal WD feeding

In 16-week-old male offspring, postnatal WD feeding alone did not significantly affect liver weight, serum alanine aminotransferase (ALT) levels and the hepatic macrophage pool. Both liver weight and serum ALT levels were significantly higher in WD/WD and WD/WD/WD mice compared with NC/WD mice, indicating liver injury (Fig. 2A and B). On flow cytometry, increasing exposure to maternal WD feeding was associated with a gradual infiltration of monocytes and depletion of Kupffer cells (KC) (Fig. 2C and Fig. S2). We then determined the NAFLD activity and fibrosis scores on histology. In NC/WD mice, only mild steatosis and inflammation were observed. In contrast, most offspring exposed to maternal WD feeding exhibited moderate steatosis with mild to moderate inflammation, resulting in a higher NAFLD activity score compared with NC/WD mice. Importantly, fibrosis (F1–F3) was only observed in offspring with exposure to maternal WD (Fig. 2F and G). Changes in both steatosis and fibrosis stages were confirmed by quantifying the liver triglyceride content and Sirius Red area, respectively (Fig. 2D and E).

Similarly, at 10 weeks of age, WD/WD mice (n = 6) exhibited increased liver weight, steatosis grade, and inflammation, but not elevated serum ALT levels, compared with NC/WD mice (n = 11). No fibrosis was observed yet. Furthermore, monocyte infiltration and KC depletion, although not significantly, were also observed (Fig. S1D–G).

In offspring at weaning age, maternal WD feeding significantly increased liver weight and serum ALT. Interestingly, moderate to severe steatosis and liver inflammation were already present at this young age (Fig. 2H).

Since MASLD is characterised by sexual dimorphism,[23] we investigated whether maternal WD feeding equally exacerbates liver disease in female offspring. This was not the case, as body and liver weight, serum ALT and liver histology were comparable between WD-fed females with (n = 9) and without (n = 6) exposure to maternal WD (Fig. S3).

Maternal hepatic IR worsens offspring inflammation and fibrosis, but not steatosis

As maternal WD feeding induces several metabolic alterations that can influence the offspring's metabolic health, we next used a genetic model to study the effect of isolated maternal hepatic IR on MASLD development in the offspring. This was achieved through a hepatocyte-specific knockout of the insulin receptor in dams. Only Cre-negative male offspring, who themselves did not have a deletion of the insulin receptor, were used in this experiment (Fig. S4A). Maternal hepatic IR did not affect body, gonadal AT or liver weight, serum ALT levels, or the hepatic macrophage pool (Fig. S4B–G). Surprisingly, offspring from hepatic IR dams (n = 9) exhibited lower fasting glucose and HOMA-IR levels compared with offspring from insulin sensitive dams (n = 12) (Fig. S4H). Contrary to steatosis, maternal hepatic IR did increase hepatic inflammation and fibrosis in the offspring (Fig. S4I and J). A comparison between the two models shows that maternal IR in itself replicates in part, but not fully, the deleterious effects of an unhealthy maternal diet.

Worsening of offspring MASLD by maternal WD feeding is characterised by mitochondrial dysfunction

As exposure to maternal WD feeding worsened offspring MASLD, we aimed to explore the underlying mechanisms of our findings. Therefore, full liver tissue transcriptomic analysis of the 16-week-old male offspring was performed. Principal component analysis (PCA) revealed four distinct clusters based on postnatal and maternal diet, and whether WD/WD and WD/WD/WD mice developed fibrosis, resulting in the following clusters: NC/NC, NC/WD, multigenerational WD, and multigenerational WD with fibrosis development (Fig. 3A). Gene Ontology (GO) biological process analysis demonstrated that among the top 10 differentially regulated pathways, four were involved in mitochondrial function, more specifically in oxidative phosphorylation (OXPHOS), when comparing NC/WD mice to mice with exposure to multigenerational WD feeding and fibrosis development (Fig. 3B). These findings were reinforced by GO cellular component and Kyoto Encyclopedia of Genes and Genomes (KEGG) pathway analysis (Fig. 3B; Fig. S5A). Additionally, genes encoding for the different subunits of the OXPHOS complexes I and V were downregulated by multigenerational WD feeding and fibrosis development (Fig. 3C).

The transcriptomic results of our mouse model were confirmed on qPCR (Table S1). Considering that OXPHOS proteins are both nuclear and mitochondrial-encoded, and pathway analysis indicated mitochondrial dysfunction by multigenerational WD feeding in our model, we focused on mitochondrial-encoded subunits, namely *Nd1*, *Cytb*, *Co1*, and *Atp6*. These subunits are part of complex I, III, IV, and V, respectively (all subunits of complex II are nuclear encoded). *Nd1* was downregulated in a stepwise fashion with increasing exposure to WD and this was further aggravated in mice with fibrosis development. The expression of *Cytb* and *Atp6* was only significantly lower in mice with fibrosis, whereas no changes in the expression of *Co1* were observed (Fig. 3D). Our data suggest that maternal WD feeding worsens MASLD and fibrosis development through downregulation of these genes.

Finally, blue-native polyacrylamide gel-electrophoresis (BN-PAGE) allowed the separation of the five OXPHOS complexes and the evaluation of their individual protein amount. Using a subsequent in-gel staining protocol, the enzyme activity of each complex could be demonstrated. WD feeding lowered the abundance and activity of complexes III, IV, and V in particular, especially in mice exposed to multigenerational WD feeding.

inflammation of the 3-week-old offspring (H). Data are presented as mean ± SD. Statistical significance was evaluated by one-way ANOVA followed by Tukey *post-hoc* testing. *p <0.05; **p <0.01; ***p <0.001; ****p <0.0001. ALT, alanine aminotransferase; KCs, Kupffer cells; MASLD, metabolic dysfunction-associated steatotic liver disease; NAFLD, non-alcoholic fatty liver disease; NAS, NAFLD activity score; NC, normal chow; WD, Western diet.

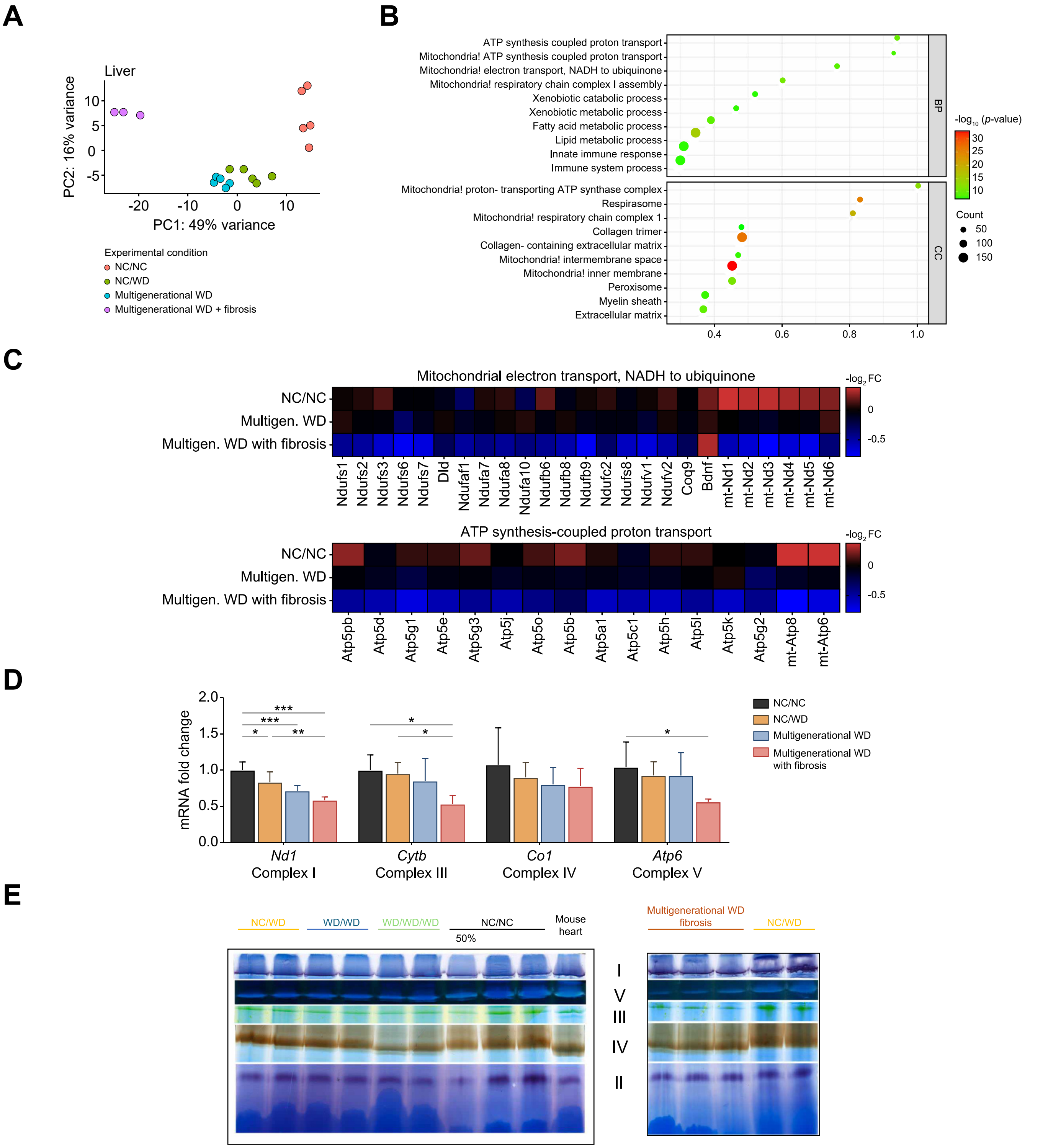

Fig. 3. Effect of maternal WD on liver transcriptomics and function of 16-week-old offspring. PCA identifying four clusters based on postnatal and maternal diet and fibrosis development (A). Top 10 differentially regulated pathways between NC/WD and multigenerational WD + fibrosis in GO BP and CC analysis, plotted with *p* value (shading), number of differentially regulated genes (circle size) and percentage of differentially regulated genes in the corresponding pathway (x-axis) (B). Heatmap of genes encoding for the different subunits of the OXPHOS complexes I and V (C). Relative gene expression levels of mitochondrial-encoded OXPHOS subunits (*Nd1*, *Cytb*, *Co1*, and *Atp6*) (D). In-gel activity staining of OXPHOS complexes with a mouse heart sample as control (E). Data are presented as mean ± SD. Statistical significance was evaluated by one-way ANOVA followed by Tukey *post-hoc* testing. **p* <0.05; ***p* <0.01; ****p* <0.001. BP, biological process; CC, cellular component; GO, Gene Ontology; NC, normal chow; PCA, principal component analysis; WD, Western diet.

The greatest reduction was observed in mice with multigenerational WD and fibrosis development (Fig. 3E).

Maternal WD feeding is associated with reduced mitochondrial content and changes in mitochondrial biogenesis, dynamics, and mitophagy

Consistent with the reduced abundance of several mitochondrial complexes on BN-PAGE, citrate synthase activity, a measure for mitochondrial content, was diminished in mice exposed to maternal WD feeding. Interestingly, mitochondrial DNA copy number was significantly elevated in mice exposed to maternal WD in absence of fibrosis, whereas it was reduced in mice with fibrosis development (Fig. 4A; Table S2). Based on the latter, mice that have not developed fibrosis (yet) may demonstrate a compensatory response to the increased FFA flux. In contrast, this adaptive mechanism appears to be attenuated in mice with fibrosis.[13]

Given the reduced content and activity of several OXPHOS complexes by maternal WD feeding, we aimed to explore the effect of maternal WD on mitochondrial biogenesis, dynamics (*i.e.* fusion and fission) and mitophagy. *Pgc1α* and *Nrf1*, both regulators of mitochondrial biogenesis, exhibited divergent responses: *Pgc1α* was significantly downregulated by WD feeding, contrary to *Nrf1*, which was significantly elevated in mice with fibrosis. The mitochondrial fusion marker *Opa1* was upregulated in mice with multigenerational WD feeding, and yet downregulated with fibrosis development. Conversely, markers of fission, *Dnm1l* and *Dnm2*, were both elevated in mice with fibrosis development. In terms of mitophagy, *Bnip3* expression was decreased in mice exposed to maternal WD, irrespective of fibrosis development. In contrast, *Parkin* tended to increase with maternal WD (Fig. 4B). These findings suggest that maternal WD feeding is associated with alterations in mitochondrial biogenesis, dynamics, and mitophagy.

We explored whether OXPHOS subunits and mitochondrial biogenesis marker expression were also affected in our model of genetic maternal IR. The complex IV subunit *Co1* and the biogenesis marker *Pgc1α* were upregulated, which is in line with the adaptive increase in mitochondrial capacity seen in more early-stage MASLD[24] (Fig. S6) – as ALT levels, steatosis, and fibrosis severity were lower than in the maternal WD model.

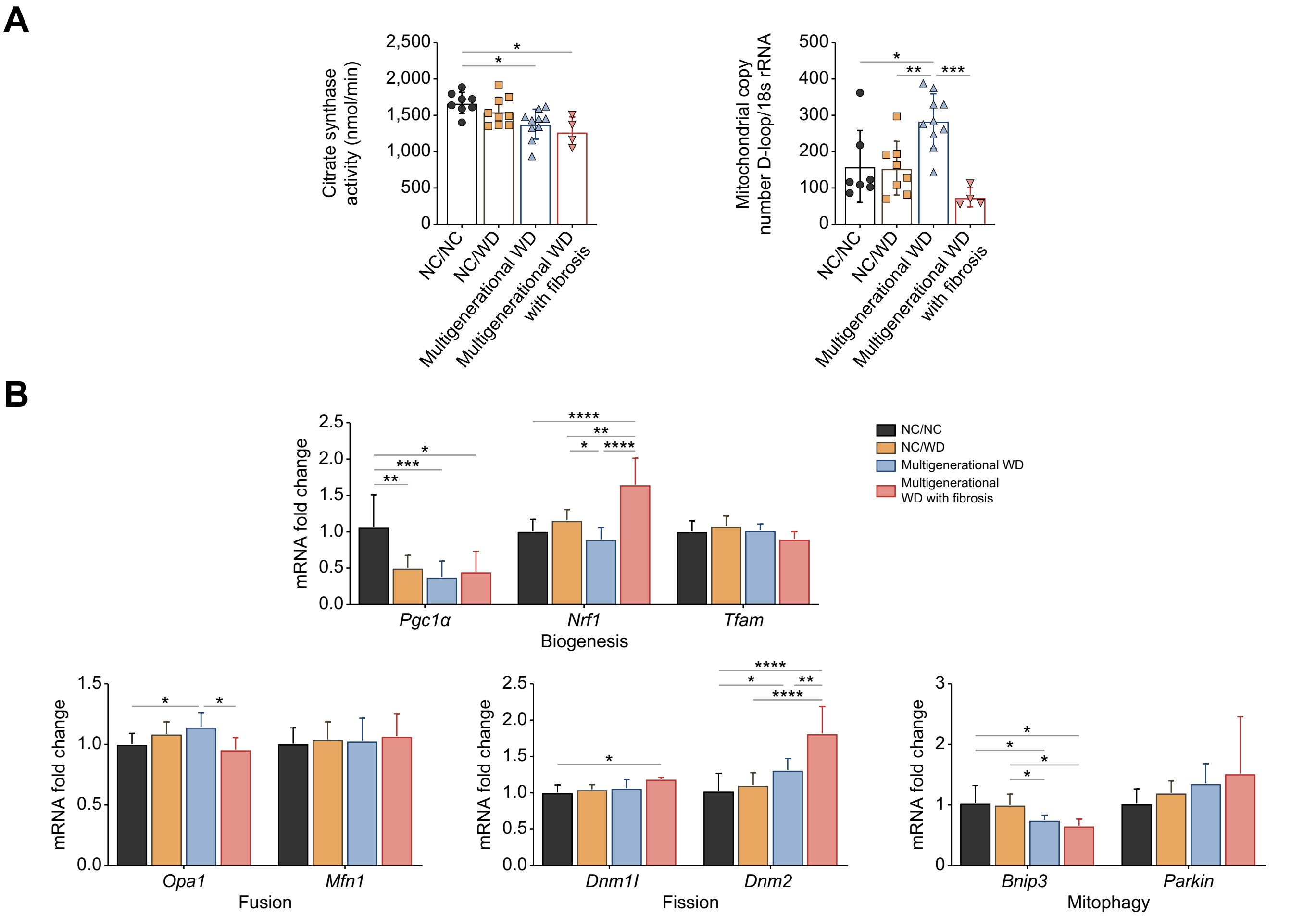

Fig. 4. Effect of maternal WD on mitochondrial function of 16-week-old offspring. Citrate synthase activity and mitochondrial copy number (A). Relative expression levels of genes involved in mitochondrial biogenesis (*Pgc1α*, *Nrf1*, *Tfam*), fusion (*Opa1* and *Mfn1*), fission (*Dnm1l* and *Dnm2*) and mitophagy (*Bnip3* and *Parkin*) (B). Data are presented as mean ± SD. Statistical significance was evaluated by one-way ANOVA followed by Tukey *post-hoc* testing. *p <0.05; **p <0.01; ***p <0.001; ****p <0.0001. NC, normal chow; WD, Western diet.

Mitochondrial function is impaired in non-human primates exposed to maternal WD and in adolescents with obesity

We then set out to investigate the translational value of our findings. First, we analysed the publicly available RNAseq dataset GSE220102.[9] The set-up of this study on maternal WD in non-human primates was similar to ours. Briefly, female Japanese macaques were either fed a NC diet or a WD before and during pregnancy and during lactation. The offspring were

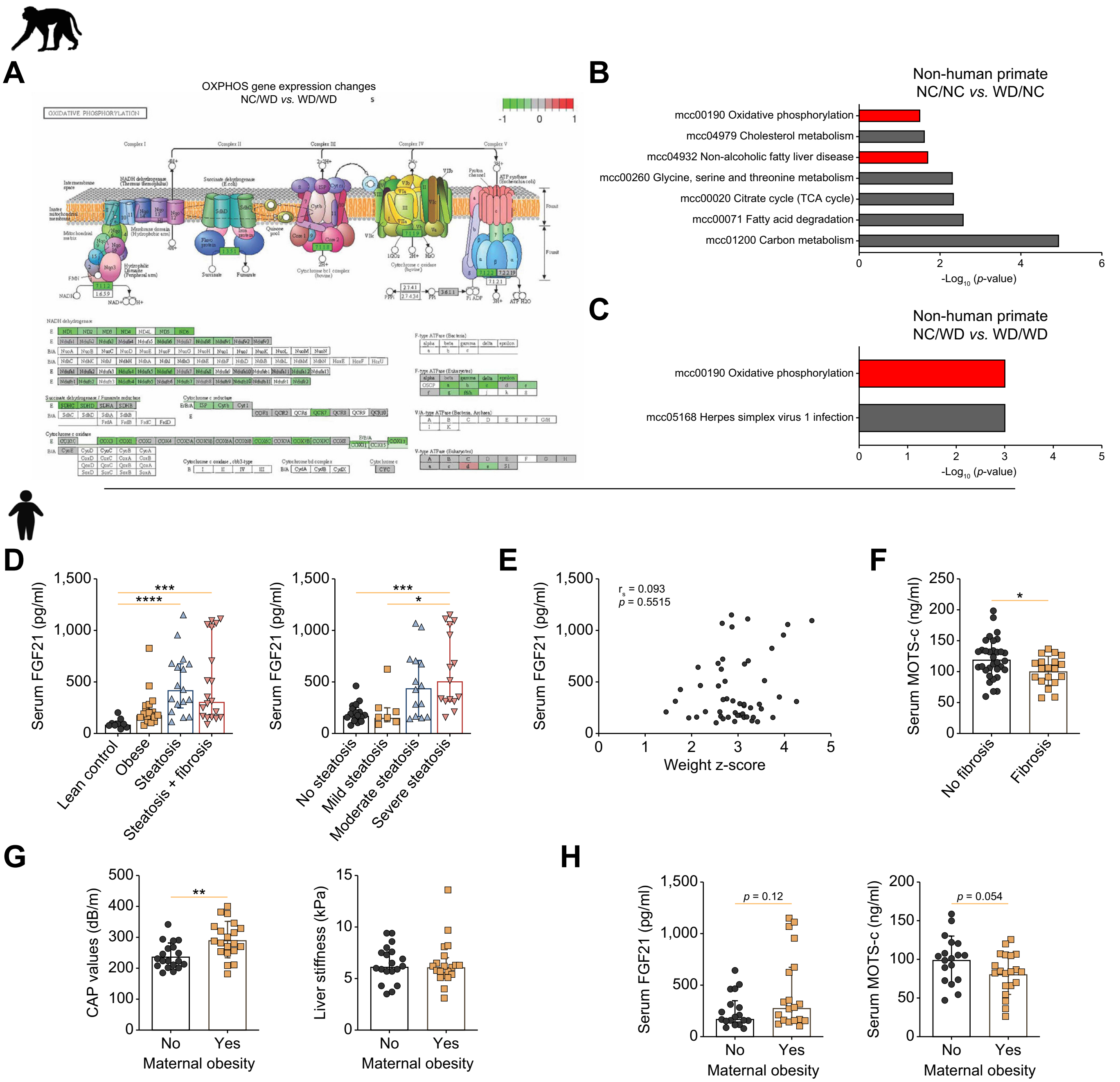

Fig. 5. Effect of maternal WD on non-human primate offspring and evaluation of serum mitochondrial biomarkers in adolescents with severe obesity and MALSD. Overview of the OXPHOS KEGG pathway indicating relative gene expression levels in WD/WD compared with NC/WD non-human primates (A). Differentially regulated pathways after KEGG pathway analysis plotted with *p* value between NC/WD and WD/WD (B) and NC/NC and WD/NC (C). Serum FGF21 levels in lean controls and patients with obesity and those with MASLD with or without fibrosis and serum FGF21 levels in lean controls and patients with obesity and worsening steatosis (D). Statistical significance was evaluated using the Kruskal–Wallis test followed by Dunn's *post-hoc* testing. Correlation between serum FGF21 levels and weight z-score (Spearman's rank correlation test) (E). Serum MOTS-c levels in patients with obesity ± fibrosis (unpaired Student *t* test) (F). CAP values (unpaired Student *t* test) and liver stiffness measurement (Mann–Whitney test) in patients with obesity ± a history of maternal obesity (G). Serum FGF21 (Mann–Whitney test) and MOTS-c (unpaired Student *t* test) levels in patients with obesity ± a history of maternal obesity (H). Normally distributed data are presented as mean ± SD, otherwise data are presented as median ± IQR. *p <0.05; **p <0.01; ***p <0.001; ****p <0.0001. CAP, controlled attenuation parameter; FGF21, fibroblast growth factor 21; KEGG, Kyoto Encyclopedia of Genes and Genomes; MASLD, metabolic dysfunction-associated steatotic liver disease; MOTS-c, mitochondrial open reading frame of 12S rRNA-c; OXPHOS, oxidative phosphorylation; WD, Western diet.

also weaned onto NC or WD and maintained on this diet until sacrifice at 3 years of age. As in our study, many genes encoding for OXPHOS subcomplexes were downregulated. KEGG pathway analysis confirmed that oxidative phosphorylation was one of only two differentially downregulated pathways when comparing WD/WD with NC/WD primates. In addition, isolated maternal WD feeding also affected this pathway (Fig. 5A–C).

Furthermore, serum biomarkers reflective of mitochondrial function, namely FGF21 and mitochondrial open reading frame of 12S rRNA-c (MOTS-c), were analysed in a cohort of adolescents with severe obesity ± MASLD, in which the prevalence of maternal obesity was high (Table 1). As discussed, FGF21 has pleiotropic actions including diminishing mitochondrial dysfunction. MOTS-c is a 16-amino-acid peptide encoded by the mitochondrial DNA and is believed to be beneficial for skeletal muscle glucose metabolism.[25] Compared with lean controls, serum FGF21 levels were increased in patients with MASLD, especially in those with severe steatosis (Fig. 5D). The correlation between serum FGF21 and MASLD severity was independent of weight, as there was no correlation between FGF21 and weight z-scores (Fig. 5E). Furthermore, serum MOTS-c levels were decreased in patients with fibrosis (Fig. 5F). Liver fat content, estimated by controlled attenuation parameter (CAP) values, but not liver stiffness, was increased in adolescents with a history of maternal obesity (Fig. 5G). Notably, there was a trend towards increase of serum FGF21 and reduction of serum MOTS-c with maternal obesity (Fig. 5H).

Diet reversal and FGF21 agonism improve maternal WD-induced MASLD

No pharmacological drug candidates for MASLD have been tested in a mouse model complicated by a maternal obesogenic diet yet. Therefore, we evaluated the effect of semaglutide, WT FGF21 and an amylin analogue in our mouse model. Female C57BL/6J breeding mice were fed a WD, and male offspring were weaned onto WD as described above. Offspring received daily semaglutide (n = 14), FGF21 (n = 12), the amylin analogue (n = 9) or vehicle (n = 12) from 8 to 16 weeks of age. As a positive control, a subset of mice were switched onto a NC diet (n = 13). Healthy controls also received daily vehicle (n = 8) (Fig. 6A). Vehicle-treated WD-fed offspring exhibited significantly increased body weight compared to the healthy controls, which was reduced by all interventions except for the amylin analogue. Furthermore, all interventions improved the gonadal AT weight to a level comparable to the healthy controls (Fig. 6B; Fig. S7A). Only semaglutide significantly improved the glucose tolerance, based on the IPGTT (Fig. 6C).

In line with our prior results, vehicle-treated WD-fed offspring developed moderate steatosis, inflammation and fibrosis, in some cases progressing to histological cirrhosis. FGF21 administration and diet reversal improved liver steatosis and inflammation almost to the point of complete resolution; in particular, diet reversal reduced fibrosis. Conversely, semaglutide and the amylin analogue did not improve steatosis, inflammation, and fibrosis (Fig. 6D). Serum ALT levels were reduced by all interventions, except semaglutide, whereas only diet reversal significantly improved *Tnfα* expression (Fig. 6E and F). Furthermore, infiltration of monocytes and monocyte-derived macrophages (MoMF) was reduced by all interventions, except semaglutide for MoMF, whereas no effect on the KC population was observed (Fig. 6G; Fig. S7C and D).

Finally, the effect of the interventions was evaluated by expression analysis of genes for mitochondrial-encoded OXPHOS subunits, mitochondrial biogenesis, dynamics, and mitophagy. *Nd1* expression was higher after diet reversal compared with semaglutide and amylin, but not FGF21 treatment. *Atp6* expression was increased by both FGF21 and amylin treatment, whereas no effect on *Cytb* and *Co1* expression was

Table 1. Characteristics of adolescents with obesity and lean controls.

Patient characteristics	Lean control (n = 9)	Obese (n = 18)	Steatosis (n = 18)	Steatosis + fibrosis (n = 19)	*p* value
Age, years (range)	13 (10–15)	16 (14–16)	16 (15–17)	16 (16–16)	0.320
Sex, female/male	3/6	11/7	11/7	10/9	0.832
Maternal obesity (no/yes)	N/A	9/5	3/10	7/5	0.073
CAP (dB/m)	173 (158-215)	213 (208–231)	311 (270–344)	298 (268–322)	**<0.001**
Steatosis (no/mild/moderate/severe), %	100/0/0/0	100/0/0/0	0/22/33/45	0/16/42/42	**<0.001**
Transient elastography, kPa	4.7 (3.5–4.9)	5.9 (4.3–6.1)	5.7 (5.4–6.2)	9.4 (8.0–10.7)	**<0.001**
Biometry					
Weight, kg	44 (40.2—55)	116.9 (107.1–121.2)	110.0 (104.6–121.9)	115.8 (107–125.3)	0.764
Weight, z-score		3.0 (2.9–3.1)	2.8 (2.6–3.4)	2.9 (2.8–3.3)	0.911
BMI, kg/m^2	19.4 (16.1-–20.3)	36.4 (35.6–41.8)	42.2 (36.6–47.4)	39.1 (36.8–45.9)	0.149
BMI, z-score	0.3 (-0.6–0.9)	2.8 (2.7–3.2)	3.0 (2.8–3.3)	2.9 (2.8–3.3)	0.704
Lab results					
ALT, U/L	14 (12–16)	19 (16–27)	25 (18–49)	32 (28–47)	**0.004**
AST, U/L	25 (18–32)	25 (20–26)	25 (20–40)	29 (25–40)	**0.048**
GGT, U/L	11 (10–14)	18 (14–23)	19(13–25)	28.5 (19–34)	**0.012**
Triglycerides, mg/dl	44 (40–83)	96 (80–115)	110 (86–145.5)	134 (92.5–166.5)	**0.049**
Glucose, mg/dl	80 (65–86)	83 (79–84)	78.5 (76–90)	81 (74–86)	0.905
Insulin, mIU/L	6.9 (3.7–8.9)	16.6 (15.0–22.7)	19.0 (14.2–42.5)	26.2 (19.4–34.9)	**0.028**
HOMA-IR	1.3 (0.6–2.1)	3.4 (2.8–5.1)	3.8 (2.7–9.6)	4.9 (3.6–7.2)	0.069
FGF21, pg/ml	91.4 (68.9–119.5)	190.1 (158.9–270.1)	480.1 (211.4–680.1)	333.3 (166.4–1,033.9)	**0.017**
MOTS-c, ng/ml		105.2 (85.9–107.8)	90.9 (83.3–106.7)	84.0 (67.8–92.6)	0.164

Continuous data are presented as the median (IQR). Statistical significance was evaluated using the Kruskal–Wallis test. Values in bold indicate significance. ALT, alanine aminotransferase; AST, aspartate aminotransferase; CAP, controlled attenuation parameter; FGF21, fibroblast growth factor 21; GGT, gamma-glutamyltransferase; HOMA-IR, homeostatic model assessment of insulin resistance; MOTS-c, mitochondrial open reading frame of 12S rRNA-c.

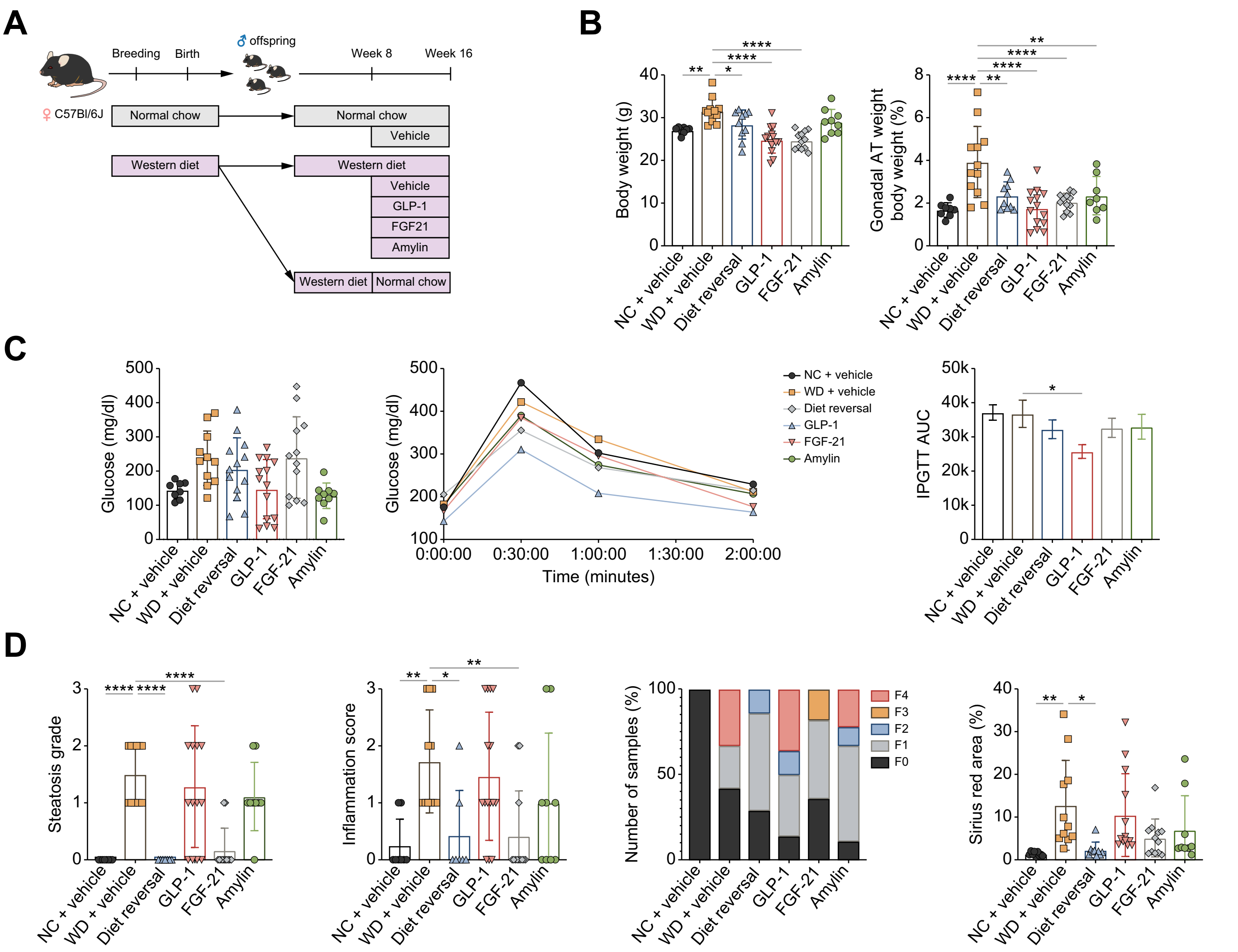

Fig. 6. Evaluation of pharmacological compounds in the maternal WD model. Schematic overview of the administration of the GLP-1 analogue, FGF21, the amylin analogue, and vehicle, in the maternal WD model. Created with BioRender (A). Body weight and relative gonadal AT weight (B). Serum glucose levels, IPGTT and AUC of IPGTT (C). Scoring of steatosis, inflammation and fibrosis and quantification of Sirius Red area (D). Serum ALT levels (E). Relative gene expression of *Tnfα* (F). Relative cell quantification of isolated monocytes and KCs (G). Relative gene expression levels of mitochondrial-encoded OXPHOS subunits (*Nd1*, *Cytb*, *Co1*, and *Atp6*) (H). Relative gene expression of *Pgc1α* (I). Data are presented as mean ± SD. Statistical significance was evaluated by one-way ANOVA followed by Tukey *post-hoc* testing. *p <0.05; **p <0.01; ***p <0.001; ****p <0.0001. ALT, alanine aminotransferase; AT, adipose tissue; FGF21, fibroblast growth factor 21; GLP-1, glucagon-like peptide 1; IPGTT, intraperitoneal glucose tolerance test; KCs, Kupffer cells; NC, normal chow; OXPHOS, oxidative phosphorylation; WD, Western diet.

observed. Diet reversal and semaglutide, but particularly FGF21 treatment, enhanced the expression of *Pgc1α*, *Nrf1* and *Bnip3* (Fig. 6H and I; Fig. S7F). In summary, FGF21 was the most promising compound to reverse both MASLD and mitochondrial dysfunction in our maternal WD model.

Discussion

Considering the increasing prevalence of maternal obesity, which was recently identified as a major health concern by the World Health Organization, investigating the effect of maternal obesity and diet on the offspring is crucial.[26] Although several studies on this topic have been performed, few have focused in depth on MASLD in the offspring. Here, we evaluated the impact of multigenerational maternal WD feeding on MASLD development in the subsequent generations. Our major findings are (1) maternal WD feeding during pregnancy and lactation induces a dysmetabolic phenotype and increased susceptibility to severe MASLD in the male offspring starting from weaning age, which is further exacerbated by multigenerational exposure; (2) the transmission of MASLD risk could partially be explained by isolated maternal hepatic IR; (3) maternal WD feeding is associated with exacerbated mitochondrial dysfunction in the offspring; (4) FGF21 agonism improves MASLD and mitochondrial dysfunction in offspring with a history of maternal WD.

Limited research on multigenerational maternal WD feeding and the effect on offspring MASLD has been performed to date.[15] In contrast, direct effects of maternal obesity and WD on offspring's health have been explored in preclinical models.[11,27–29] The general conclusion of these studies is that maternal obesity aggravates offspring metabolic disease, liver steatosis, and fibrosis. In addition, several studies demonstrated

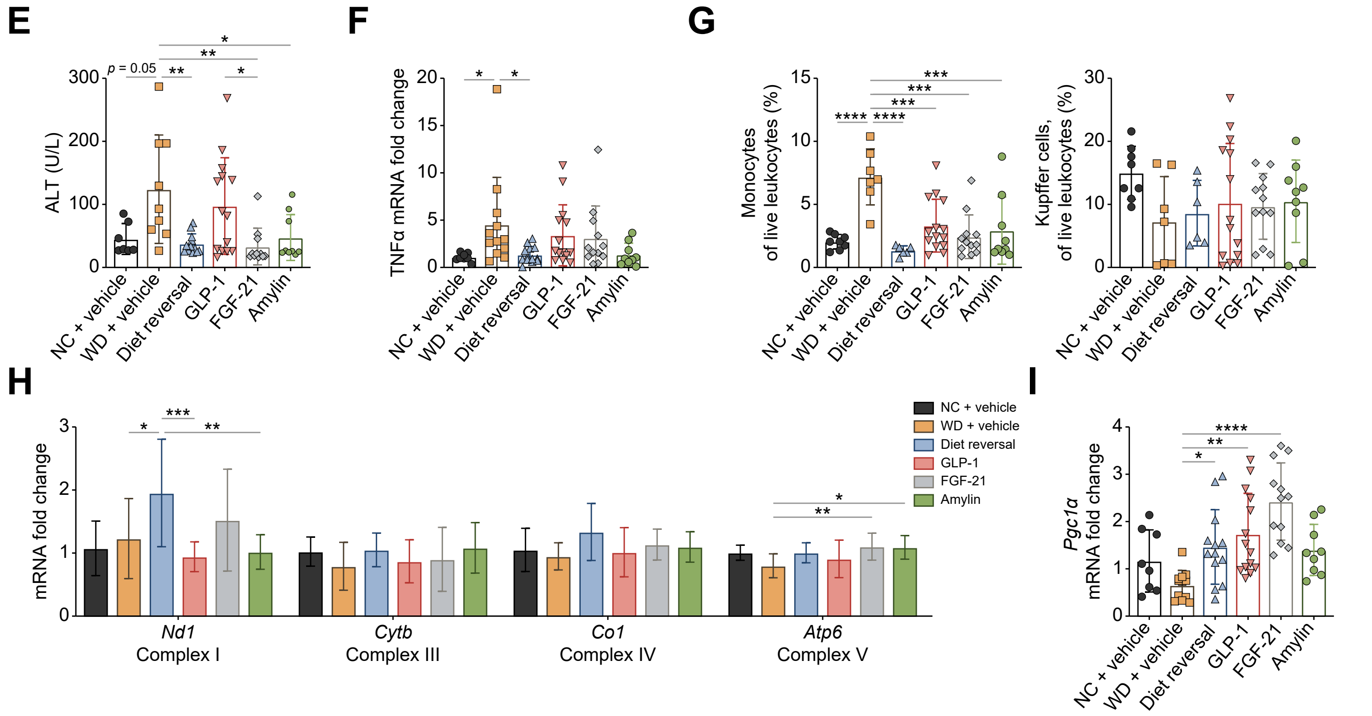

Fig. 6. (*continued*).

a dysmetabolic phenotype and MASLD in offspring with maternal obesity that were weaned onto a control diet.[11,28] These results are consistent with our data, as we found that offspring exposed to (multigenerational) maternal WD feeding already developed hepatic steatosis and inflammation at weaning age. This highlights the importance of *in utero* and early life insults and aligns with the Developmental Origins of Health and Disease. However, Thompson *et al.*[29] found a protective effect against severe MASLD by the initial maternal high fat/high sucrose diet, as fibrosis was decreased in the next three generationsKlik of tik om tekst in te voeren. Interestingly, although we observed a significant exacerbation of metabolic phenotype and MASLD in offspring exposed to maternal WD compared with those without exposure, the differences between exposure to one *vs*. two generations of maternal WD were markedly less pronounced. Specifically, two generations of maternal WD did exacerbate the dysmetabolic phenotype and altered the macrophage pool in the offspring. Conversely, hepatic steatosis, inflammation, and fibrosis on histology were not aggravated in offspring exposed to two generations of maternal WD. The worse metabolic phenotype observed in these mice may result in more severe MASLD when WD feeding is prolonged. Additional studies are required to further elaborate on these findings.

Furthermore, our findings could not be reproduced in female offspring, which is consistent with previous studies indicating that female C57BL/6 mice are more resistant to develop a dysmetabolic phenotype.[30] Whereas de Jesus and colleagues[8] reported that isolated maternal hepatic IR promoted offspring body weight gain and IR, we could not replicate this finding. Nevertheless, liver inflammation and fibrosis were aggravated, albeit to a lesser extent when compared with the offspring of WD-fed mothers. This seems reasonable, as the latter induces not only hepatic, but whole-body IR.

In line with the pathogenesis in patients, mitochondrial dysfunction was identified as a key mechanism to worsen MASLD in our model. It is well established that mitochondrial dysfunction, through oxidative stress and reactive oxygen species production, plays a pivotal role in the progression to MASH and fibrosis development.[24,31] However, the impact of maternal WD consumption on mitochondrial biogenesis, dynamics, and mitophagy is less clear. In human MASH, markers of mitochondrial biogenesis (e.g. PGC1α, NRF1, TFAM),[13] are downregulated, which we also found regarding *Pgc1α*. The prevailing view is that mitochondria are initially able to adapt to an increased load of hepatic fatty acids by upregulating OXPHOS capacity, at the cost of producing oxidative stress. The progression of MASH and fibrosis then coincides with a downturn in mitochondrial function, which is associated with alterations in fusion–fission cycles and mitophagy. Indeed, we found that mice with fibrosis development exhibited reduced expression of fusion and mitophagy markers. Notably, Moore *et al.*[32] demonstrated similar mitochondrial alterations on liver biopsies of patients with worsening steatotic liver disease.Klik of tik om tekst in te voeren. Mechanistically, our data in the mouse models, and vindicated by analysis of non-human primates, indicate that maternal WDconsumption can be a first hit to mitochondrial OXPHOS capacity specifically, predisposing towards more rapid disease progression in the offspring. Supporting this hypothesis, serum FGF21 and MOTS-c levels were dysregulated depending on MASLD severity in a cohort of adolescents characterised by a high prevalence of maternal obesity.

For the first time, we evaluated promising pharmacological compounds in a maternal WD mouse model. Since maternal WD can induce epigenetic changes and alter the microbiome in the offspring, the efficacy of compounds can be decreased in patients with a history of a maternal dysmetabolic phenotype.

As a positive control, we incorporated a diet reversal group. Although resmetirom was just recently approved by the FDA for the therapy of fibrotic MASH, lifestyle intervention is still the cornerstone of treatment. Many studies show a high impact of weight loss and diet change on MASLD.[33–35] We found similar results, as only 8 weeks on a control diet resulted in a significant improvement of MASLD severity and prevention of fibrosis development. Importantly, compared with humans, mice lose weight and recover from MASH more quickly after dietary intervention.[36] Furthermore, with the obesogenic environments in which we currently live and potential increased resistance to weight loss through epigenetic changes,[37] evaluation of pharmacological therapy is essential.

Phase II clinical trials involving FGF21 agonists have demonstrated improvement of liver fibrosis and resolution of MASH.[19] These findings may appear counterintuitive, because several studies, including ours, have shown a positive correlation between serum FGF21 levels and MASLD severity.[38,39] This paradox can be explained by the phenomenon of FGF21 resistance, arising from the downregulation of the co-receptor beta-klotho, which is observed in patients with chronic metabolic dysfunction. This results in a compensatory upregulation of the production and secretion of FGF21, thereby increasing its serum levels.[40] In our study, compared with the two other compounds tested, FGF21 agonism showed the greatest amelioration of MASLD. Importantly, FGF21 is the only compound tested with known hepatic expression of receptors. Furthermore, one mechanism of action of FGF21 is improvement of mitochondrial function and oxidative stress, which results in less cellular damage and increased FAO.[18] With mitochondrial dysfunction being one of the contributors of disease severity in our mouse model, the promising results of FGF21 in the offspring are likely to be caused by improvement of mitochondrial function. Indeed, FGF21 agonism upregulated genes involved in mitochondrial biogenesis. Conversely, semaglutide and amylin agonism did not improve MASLD in our study. This may be attributed to the absence of hepatic receptors and thus on direct effects on mitochondrial function, instead relying on indirect effects by treating obesity.[41]

In conclusion, we showed that multigenerational maternal WD aggravates MASLD in male offspring starting from weaning age with mitochondrial dysfunction contributing to disease severity. FGF21 agonism, in particular, improved MASLD in offspring exposed to maternal WD.

Affiliations

[1]*Liver Research Center Ghent, Ghent University, Ghent University Hospital, Ghent, Belgium;* [2]*Department of Internal Medicine and Pediatrics, Hepatology Research Unit, Ghent University, Ghent, Belgium;* [3]*Department of Basic and Applied Medical Sciences, Gut-Liver Immunopharmacology Unit, Ghent University, Ghent, Belgium;* [4]*Translational Nuclear Receptor Research, Department of Biomolecular Medicine, VIB Center for Medical Biotechnology, Ghent, Belgium;* [5]*Department of Human Structure and Repair, Department of Gastrointestinal Surgery, Ghent University, Ghent, Belgium;* [6]*Department of Pharmaceutics, Laboratory of Pharmaceutical Biotechnology, Ghent University, Ghent, Belgium;* [7]*Department of Internal Medicine and Pediatrics, Division of Pediatric Neurology and Metabolism, Ghent University, Ghent, Belgium;* [8]*Zeepreventorium, De Haan, Belgium;* [9]*Department of Internal Medicine and Pediatrics, Pediatric Gastroenterology, Hepatology and Nutrition, Ghent University, Ghent, Belgium*

Abbreviations

ALT, alanine aminotransferase; AST, aspartate aminotransferase; AT, adipose tissue; BN-PAGE, blue-native polyacrylamide gel-electrophoresis; BP, biological process; CAP, controlled attenuation parameter; CC, cellular component; FAO, fatty acid oxidation; FGF21, fibroblast growth factor 21; GGT, gamma-glutamyl transferase; GLP-1, glucagon-like peptide 1; GO, gene ontology; HOMA-IR, homeostatic model assessment of insulin resistance; IPGTT, intraperitoneal glucose tolerance test; IR, insulin resistance; KCs, Kupffer cells; KEGG, Kyoto Encyclopedia of Genes and Genomes; LIRKO, liver-specific insulin receptor knockout; MASH, metabolic-associated steatohepatitis; MASLD, metabolic dysfunction-associated steatotic liver disease; MoMF, monocyte-derived macrophages; MOTS-c, mitochondrial open reading frame of 12S rRNA-c; NAFLD, non-alcoholic fatty liver disease; NC, normal chow; OXPHOS, oxidative phosphorylation; PCA, principal component analysis; WD, Western diet.

Financial support

This work is supported by a grant from the Ghent University Hospital (FIKO19-TYPE2-006). SL and MA are supported by the Research Foundation – Flanders (FWO) (1227824N and 11B0723N). SR, RDB, and AG are senior clinical investigators of the FWO (1802624N, 1843824N, and 1805718N). AV is a clinical researcher supported by the Ghent University by a BOF-Tenure Track (BOF/STA/202209/040). These funding agencies were not involved in study design, analysis or reporting.

Conflicts of interest

The authors have no conflicts of interest to report.

Please refer to the accompanying ICMJE disclosure forms for further details.

Authors' contributions

Conceptualisation: SL, AG, RDB, AH. Formal analysis: AH, MA, LO, SL, ED, FVN, YG, TM, JS, AV. Investigation: AH, MA, LO, SL, ED, FVN, YG, TM, JS, AV. Methodology: SL, AG, RDB, AH, LD, HVV, XV, SR. Supervision: SL, AG, RDB. Writing – original draft: AH. Writing – review and editing: SL, AH, HVV, XV, SR, LD. All authors reviewed and approved the manuscript.

Data availability statement

RNA sequencing data are publicly available via the NCBI repository under the number GSE291363. Other data are available by reasonable request to the authors.

Acknowledgements

We cordially thank Petra Van Wassenhove, Els Van Deynse, and Inge Van Colen for their excellent technical support, and the team of Zeepreventorium for their enthusiastic participation in this study.

Supplementary data

Supplementary data to this article can be found online at https://doi.org/10.1016/j.jhepr.2025.101404.

References

Author names in bold designate shared co-first authorship

[1] Anderson EL, Howe LD, Jones HE, et al. The prevalence of non-alcoholic fatty liver disease in children and adolescents: a systematic review and meta-analysis. PLoS One 2015;10:e0140908.

[2] Younossi ZM, Golabi P, Paik JM, et al. The global epidemiology of nonalcoholic fatty liver disease (NAFLD) and nonalcoholic steatohepatitis (NASH): a systematic review. Hepatology 2023;77:1335–1347.

[3] **Hagström H**, **Simon TG**, Roelstraete B, et al. Maternal obesity increases the risk and severity of NAFLD in offspring. J Hepatol 2021;75:1042–1048.

[4] Hagström H, Tynelius P, Rasmussen F. High BMI in late adolescence predicts future severe liver disease and hepatocellular carcinoma: a national, population-based cohort study in 1.2 million men. Gut 2018;67:1536–1542.

[5] Querter I, Pauwels NS, De Bruyne R, et al. Maternal and perinatal risk factors for pediatric nonalcoholic fatty liver disease: a systematic review. Clin Gastroenterol Hepatol 2022;20:740–755.

[6] Strauss A, Rochow N, Kunze M, et al. Obesity in pregnant women: a 20-year analysis of the German experience. Eur J Clin Nutr 2021;75:1757–1763.

[7] Orós M, Lorenzo M, Serna MC, et al. Obesity in pregnancy as a risk factor in maternal and child health—a retrospective cohort study. Metabolites 2024; 14:56.
[8] de Jesus DF, Orime K, Kaminska D, et al. Parental metabolic syndrome epigenetically reprograms offspring hepatic lipid metabolism in mice. J Clin Invest 2020;130:2391–2404.
[9] Nash MJ, Dobrinskikh E, Janssen RC, et al. Maternal Western diet is associated with distinct preclinical pediatric NAFLD phenotypes in juvenile nonhuman primate offspring. Hepatol Commun 2023;7. https://doi.org/10.1097/hc9.0000000000000014. e0014–e0014.
[10] Bruce KD, Cagampang FR, Argenton M, et al. Maternal high-fat feeding primes steatohepatitis in adult mice offspring, involving mitochondrial dysfunction and altered lipogenesis gene expression. Hepatology 2009;50: 1796–1808.
[11] Thompson MD, Cismowski MJ, Trask AJ, et al. Enhanced steatosis and fibrosis in liver of adult offspring exposed to maternal high-fat diet. Gene Expr 2016;17:47–59. https://doi.org/10.3727/105221616X692135.
[12] Shami GJ, Cheng D, Verhaegh P, et al. Three-dimensional ultrastructure of giant mitochondria in human non-alcoholic fatty liver disease. Sci Rep 2021;11:3319.
[13] Koliaki C, Szendroedi J, Kaul K, et al. Adaptation of hepatic mitochondrial function in humans with non-alcoholic fatty liver is lost in steatohepatitis. Cel Metab 2015;21:739–746. https://doi.org/10.1016/j.cmet.2015.04.004.
[14] **Lee W**, **Zamudio-Ochoa A**, **Buchel G**, et al. Molecular basis for maternal inheritance of human mitochondrial DNA. Nat Genet 2023;55:1632–1639.
[15] Li J, Huang J, Li J-S, et al. Accumulation of endoplasmic reticulum stress and lipogenesis in the liver through generational effects of high fat diets. J Hepatol 2012;56:900–907.
[16] Lee HA, Kim HY. Therapeutic mechanisms and clinical effects of glucagon-like peptide 1 receptor agonists in nonalcoholic fatty liver disease. Int J Mol Sci 2023;24:9324.
[17] Newsome PN, Buchholtz K, Cusi K, et al. A placebo-controlled trial of subcutaneous semaglutide in nonalcoholic steatohepatitis. New Engl J Med 2021;384:1113–1124.
[18] **Lee JH**, **Kang YE**, Chang JY, et al. An engineered FGF21 variant, LY2405319, can prevent non-alcoholic steatohepatitis by enhancing hepatic mitochondrial function. Am J Transl Res 2016;8:4750–4763.
[19] Loomba R, Sanyal AJ, Kowdley KV, et al. Randomized, controlled trial of the FGF21 analogue pegozafermin in NASH. New Engl J Med 2023;389: 998–1008.
[20] Harrison SA, Frias JP, Neff G, et al. Safety and efficacy of once-weekly efruxifermin versus placebo in non-alcoholic steatohepatitis (HARMONY): a multicentre, randomised, double-blind, placebo-controlled, phase 2b trial. Lancet Gastroenterol Hepatol 2023;8:1080–1093.
[21] Li C, Xu JJ, Hu HT, et al. Amylin receptor insensitivity impairs hypothalamic POMC neuron differentiation in the male offspring of maternal high-fat diet-fed mice. Mol Metab 2021;44:101135.
[22] Gydesen S, Hjuler ST, Freving Z, et al. A novel dual amylin and calcitonin receptor agonist, KBP-089, induces weight loss through a reduction in fat, but not lean mass, while improving food preference. Br J Pharmacol 2017;174:591–602.
[23] Ayonrinde OT, Adams LA, Mori TA, et al. Sex differences between parental pregnancy characteristics and nonalcoholic fatty liver disease in adolescents. Hepatology 2018;67:108–122.
24 Fromenty B, Roden M. Mitochondrial alterations in fatty liver diseases. J Hepatol 2023;78:415–429.
25 Yin Y, Pan Y, He J, et al. The mitochondrial-derived peptide MOTS-c relieves hyperglycemia and insulin resistance in gestational diabetes mellitus. Pharmacol Res 2022;175.
26 World Health Organization. Regional office for europe. WHO European regional obesity: report. Geneva: WHO; 2022.
27 Mouralidarane A, Soeda J, Visconti-Pugmire C, et al. Maternal obesity programs offspring nonalcoholic fatty liver disease by innate immune dysfunction in mice. Hepatology 2013;58:128–138.
28 Oben JA, Mouralidarane A, Samuelsson AM, et al. Maternal obesity during pregnancy and lactation programs the development of offspring nonalcoholic fatty liver disease in mice. J Hepatol 2010;52:913–920.
29 Thompson MD, Derse A, Ferey JLA, et al. Transgenerational impact of maternal obesogenic diet on offspring bile acid homeostasis and nonalcoholic fatty liver disease. Am J Physiol Endocrinol Metab 2019;316:674–686.
30 de Souza GO, Wasinski F, Donato J. Characterization of the metabolic differences between male and female C57BL/6 mice. Life Sci 2022;301:120636.
31 García-Ruiz C, Fernández-Checa JC. Mitochondrial oxidative stress and antioxidants balance in fatty liver disease. Hepatol Commun 2018;2:1425–1439.
32 Moore MP, Cunningham RP, Meers GM, et al. Compromised hepatic mitochondrial fatty acid oxidation and reduced markers of mitochondrial turnover in human NAFLD. Hepatology 2022;76:1452–1465.
33 Schwimmer JB, Ugalde-Nicalo P, Welsh JA, et al. Effect of a low free sugar diet vs usual diet on nonalcoholic fatty liver disease in adolescent boys: a randomized clinical trial. JAMA 2019;321:256–265.
34 Vilar-Gomez E, Martinez-Perez Y, Calzadilla-Bertot L, et al. Weight loss through lifestyle modification significantly reduces features of nonalcoholic steatohepatitis. Gastroenterology 2015;149. 367–78.e5.
35 Patel NS, Doycheva I, Peterson MR, et al. Effect of weight loss on magnetic resonance imaging estimation of liver fat and volume in patients with nonalcoholic steatohepatitis. Clin Gastroenterol Hepatol 2015;13. 561–68.e1.
36 Flensted-Jensen M, Oró D, Rørbeck EA, et al. Dietary intervention reverses molecular markers of hepatocellular senescence in the GAN diet-induced obese and biopsy-confirmed mouse model of NASH. BMC Gastroenterol 2024;24:59.
37 Mahmoud AM. An overview of epigenetics in obesity: the role of lifestyle and therapeutic interventions. Int J Mol Sci 2022;23:1341.
38 Barb D, Bril F, Kalavalapalli S, et al. Plasma fibroblast growth factor 21 is associated with severity of nonalcoholic steatohepatitis in patients with obesity and type 2 diabetes. J Clin Endocrinol Metab 2019;104:3327–3336.
39 Ajaz S, McPhail MJ, Gnudi L, et al. Mitochondrial dysfunction as a mechanistic biomarker in patients with non-alcoholic fatty liver disease (NAFLD). Mitochondrion 2021;57:119–130.
40 Harrison SA, Rolph T, Knott M, et al. FGF21 agonists: an emerging therapeutic for metabolic dysfunction-associated steatohepatitis and beyond. J Hepatol 2024;81:562–576.
41 Newsome PN, Ambery P. Incretins (GLP-1 receptor agonists and dual/triple agonists) and the liver. J Hepatol 2023;79:1557–1565.

Keywords: MASH; NAFLD; FGF21; Oxidative phosphorylation.
Received 27 January 2025; received in revised form 14 March 2025; accepted 21 March 2025; Available online 29 March 2025

Journal of Hepatology, Volume 7

Supplemental information

Mitochondrial dysfunction characterises the multigenerational effects of maternal obesity on MASLD

Anneleen Heldens, Milton Antwi, Louis Onghena, Tim Meese, Yannick Gansemans, Joél Smet, Ellen Dupont, Xavier Verhelst, Sarah Raevens, Hans Van Vlierberghe, Arnaud Vanlander, Filip Van Nieuwerburgh, Lindsey Devisscher, Ruth De Bruyne, Anja Geerts, and Sander Lefere

Mitochondrial dysfunction characterises the multigenerational effects of maternal obesity on MASLD

Anneleen Heldens, Milton Antwi, Louis Onghena, Tim Meese, Yannick Gansemans, Joél Smet, Ellen Dupont, Xavier Verhelst, Sarah Raevens, Hans Van Vlierberghe, Arnaud Vanlander, Filip Van Nieuwerburgh, Lindsey Devisscher, Ruth De Bruyne, Anja Geerts, Sander Lefere

Table of contents

Supplementary materials and methods……………………………………………..2

Supplementary tables...…12

Supplementary figures ....……14

Supplementary references ....……21

Supplementary materials and methods

Tissue sampling

Before sacrifice, mice were anesthetized with xylazine (10mg/kg; Sedaxyl) and ketamine (100mg/kg; Nimatek). After taken blood samples retro-orbitally, mice were euthanized via cervical dislocation. Liver, spleen and gonadal adipose tissue were weighed. Specific liver lobes were isolated for histology, gene expression analysis and flow cytometry.

Histology

Part of the liver were fixed in 4% paraformaldehyde (VWR, Avantor, Leuven, Belgium) for 24h and subsequently embedded in paraffin. Tissues were sectioned at 4µm using a Leica RM2145 microtome (Leica Biosystems, Diegem, Belgium). Liver sections were stained with hematoxylin-eosin (H&E) (Sigma-Aldrich, Overijse, Belgium) and Sirius red (SR) (Sigma-Aldrich) to evaluate MASLD severity and fibrosis respectively. MASLD severity was assessed according to the NAFLD activity score which considers steatosis, hepatocyte ballooning and lobular inflammation. Additionally, Sirius red area was evaluated according to the NAFLD clinical research network fibrosis scoring system and quantified using ImageJ software.

Serum analysis

Blood samples were centrifuged at 4°C. Serum was stored at -80°C before determining alanine aminotransferase (ALT), uric acid and glucose levels (UV test at 37°C; Roche Modular pre-analytics system, Rotkreuz, Switzerland). Serum insulin

levels were determined by enzyme-linked immunosorbent assay (EZRMI-13K; Merck, Overijse, Belgium) according to the manufacturer's instructions.

Intraperitoneal glucose tolerance test

An intraperitoneal glucose tolerance test (IPGTT) was performed seven days before sacrifice. Mice were fasted for 5h prior to intraperitoneal injection of glucose (2g/kg dissolved in 0.9% NaCl; Sigma-Aldrich). Blood glucose was measured in the tail vein with a glucometer (Bayer Contour Next, Basel, Switzerland) at baseline and 30, 60 and 120 min after glucose injection.

Flow cytometry and fluorescence activated cell sorting

The left liver lobe was perfused with cold phosphate buffered saline (PBS; Gibco, Thermo Fisher Scientific, Merelbeke, Belgium) and dissociated enzymatically through incubation with 1mg/mL collagenase A (Sigma-Aldrich) and 300µg/mL DNAse I (Sigma-Aldrich) at 37°C, and mechanically using the gentleMACS dissociator (Miltenyi Biotec, Leiden, The Netherlands). Prestaining of the single cell suspension was performed using Zombie Aqua (Fixable Viability Dye; Biolegend, London, United Kingdom), Trustain FcX Plus (anti-mouse CD16/32) and True-Stain monocyte blocker (Biolegend). Subsequently, cells were stained with CD31-PE/Dazzle 594, Clec4F-AF647, F4/80-FITC, Ly6C-BV650, Ly6G-BV785 (Biolegend), CD11b-PE-Cy7, CD45-APC-Cy7, SiglecF-PerCP-Cy5.5 and Tim4-PE (BD Biosciences, Erembodegem, Belgium) and analyzed with a BD FACSAria Fusion flow cytometer (BD Biosciences) and FlowJo software (FlowJo LLC, BD Biosciences). Absolute cell numbers were calculated using Precision Count Beads (Biolegend). After selection of live $CD45^+$ single cells, neutrophils were gated as $Ly6G^+$, monocytes as $Ly6G^-$ $CD11b^+$ $Ly6C^{hi}$,

MoKCs as Ly6G$^-$ CD11b$^+$ Ly6C^{lo} Clec4F$^+$ Tim4$^-$, KCs as Ly6G$^-$ CD11b$^+$ Ly6C^{lo} Clec4F$^+$ Tim4$^+$, and MoMfs as Ly6G$^-$ CD11b$^+$ Ly6C^{lo} Clec4F$^-$ Tim4$^-$ F4/80$^+$ SiglecF$^-$ SSC^{lo}.

Hepatic triglyceride assay

Snapfrozen liver tissue was homogenized in IGEPAL CA-360 (Sigma-Aldrich) using the TissueLyser LT (Qiagen, Hilden, Germany). Shaking was performed at 50Hz for 1 and subsequently 2 minutes. Triglyceride content was measured using the Triglyceride quantification kit (MAK266; Sigma-Aldrich) according to the manufacturer's instructions. The absorbance was determined at 570nm using Ascent software version 2.6 (Thermo Fisher Scientific).

Genotyping LIRKO mice

The genotype of breeding mice and male offspring was determined on tail or ear biopsies. DNA was extracted by boiling the tissue sample in PCR buffer (25mM NaOH and 0.2mM EDTA in distilled water; pH = 8). PCR was performed using the Biotaq DNA polymerase kit (Bioline, London, United Kingdom), dNTP mix (bioline) and the following primers: forward insulinreceptor floxed primer 5'-GGGGCAGTCAGTATTTTGGA-3', Reverse insulin receptor floxed primer 5'-TGGCCGTGAAAGTTAAGAGG-3'. For determining Cre genotype, the following primers were used: 5'-CGCCGTAAATCAATCGATGAGTTGCTTS-3', 5'-GATGCCGGTGAACGTGCAAAACAGGCTC-3', 5'-CAAACCTGCTACCCGAACCT-3', and 5'-CAGTATGCGGAAGTTCTAGG-3'. This results in a 145bp band for IR floxed mice and a 105bp band for wild type mice. The presence of the Cre allele results in a 500bp band.

RNA isolation and quantitative PCR (qPCR)

After homogenization of 20mg full liver tissue with the TissueLyser LT (Qiagen) as described above, RNA was extracted using the Aurum Total RNA Mini Kit (Bio-rad Laboratories, Temse, Belgium) according to the manufacturer's instructions. RNA concentration and purity was measured by spectrophotometry (Nanodrop ND-1000; Thermo Fisher Scientific). cDNA was obtained using the SensiFast cDNA Synthesis Kit (Bioline) according to the manufacturer's instructions, after dilution of RNA samples to a concentration of 100ng/µL with RNase-free water. Analysis was performed on the LightCycler 480 (Roche, Vilvoorde, Belgium) using diluted cDNA (1/10), SYBR Green mix (Sensimix; Bioline) and specific mouse primers (Bioline) (Table S1). Samples were measured in duplicate. Reactions were normalized with the following household genes: *Hmbs*, *Hprt* and *Gapdh*. Cq values were calculated with the second derivative maximum method.

RNA sequencing

Full liver tissue from the following 16-week-old offspring was sequenced: NC-fed (n = 5) and WD-fed (n = 5) offspring without maternal obesity and WD-fed offspring with maternal obesity with (n = 3) and without (n = 5) fibrosis development. RNA integrity of the samples was checked using the RNA 6000 Pico Kit on a Bioanalyer (Agilent Technologies Inc, Santa Clara, CA, USA). Concentration of input RNA was measured using the Quant-iT RiboGreen RNA Assay Kit (Invitrogen Inc, Waltham, MA, USA). A sequencing library was constructed for each sample using 500 ng of input RNA with the QuantSeq 3' mRNA-Seq Library Prep Kit FWD for Illumina (Lexogen Inc, Greenland, NH, USA) and the UMI Second Strand Synthesis Module for QuantSeq FWD (Illumina Inc, San Diego, CA, USA) to incorporate a 6 nt unique molecular

identifier (UMI) present at the 5'-end of the read after sequencing. Library enrichment was done with 13 PCR cycles and clean-up was done using the kit's PB beads. Quality control of the libraries was done on a Bioanalyzer using a High Sensitivity DNA Kit (Agilent Technologies Inc, Santa Clara, CA, USA) and via qPCR according to the Illumina Sequencing Library qPCR Quantification Guide. Finally, the libraries were spiked with 20% PhiX (Illumina Inc, San Diego, CA, USA) and sequenced as single-read 76 on a NextSeq 500 device (Illumina, San Diego, CA, USA).

Differential expression analysis of RNAseq data

Quality control of the raw sequencing reads was done with FastQC (v0.11.9). Contamination was checked using FastQ Screen (v0.15.1) and genomes from a limited set of common lab organisms. We used UMI-tools (v1.1.2) to remove the Unique molecular identifier (UMI) from the read sequence and add it to the read name. Adapter and quality trimming was done with cutadapt (v4.1) using default parameters with the additional use of a phred score threshold of 20. The trimmed reads were mapped on the mouse genome (GRCm39, ENSEMBL release 104) using the splice aware STAR (v2.7.10a) mapper and sorted using samtools (v1.6). Removal of mapped PCR duplicated reads was done with UMI-tools (v1.1.2), using the UMI and mapping information. Expression counts at the gene level were generated with rsem-calculate-expression (RSEM v1.36.1). To estimate if sequencing depth was reflecting the sample's complexity, we collected data for saturation plots using preseq (v3.1.1). Additional QC was done using RseQC (v4.0.0). All statistical analyses were done in R (v4.2.1) using the DESeq2's (v1.36.0) package for differential expression analysis. Multiple testing correction of p values was done using the Benjamini-Hochberg method. Differentially expressed features were considered significant when having a

fold change reflecting at least a doubling or halving (i.e., the absolute value of log2FC ≥ 1), and a corrected p value ≤ 0.05. Data were deposited to the NCBI GEO database under the accession number GSE291363.

Differential expression analysis of NCBI GEO RNAseq data from non-human primate liver samples

Raw sequencing reads, experimental design and sample metadata were downloaded from NCBI GEO accession GSE220102. The data set consisted of 33 samples in 4 treatment groups.

Raw sequencing reads were inspected using FastQC (v0.12.1) for their quality and length. Putative contaminations were checked using FastQ Screen (v0.15.3) and a set of genomes of common lab organisms. Adaptor trimming was done using cutadapt (v4.4) with added filtering of reads containing ambiguities or not passing the phred score threshold of 20. For each sample, trimmed reads were mapped on the Macaca mulatta genome (Macaca_mulatta.Mmul_10, ENSEMBL release 109) using the splice-aware STAR (v2.7.10a) mapper. UMI-based deduplication of mapped reads was done with UMI-tools (v1.1.4). Feature counting at the gene and transcript isoform level was done using rsem-calculate-expression (RSEM v1.3.3). All statistical analyses were done in R (v4.2.2) and pairwise comparisons of treatments were done using the edgeR (v3.38.4) package. Correction of the p values for repeated testing (PAdj) was done with the Benjamini-Hochberg method. Differentially expressed features were considered significant when having a fold change reflecting at least a doubling or halving (i.e., the absolute value of log2FC ≥1), and a corrected p value ≤0.05. Pathway enrichment analysis was done in R using the GAGE package (v2.44.0)

with KEGG Macaca mulatta pathway data. GO term enrichment analysis was also done with the GAGE package.

Blue-native polyacrylamide gel-electrophoresis (BN-PAGE) and in-gel activity staining of OXPHOS complexes

Mitochondria were isolated from 50mg of liver tissue. First, liver tissue with 19 volumes of ice cold mitochondrial isolation buffer (10mM Tris-HCl, 0.25M sucrose, 2mM EDTA and 50U/mL heparin, pH 7.4) was minced using scissors. Homogenization of the tissue was performed using a motor driven glass pestle with application of 20 strokes at 800rpm. Subsequently, the homogenates were sonicated for 2 seconds at medium intensity and centrifugated at 5600g for 2 minutes. The supernatant with mitochondria was kept on ice, while the pellet with remaining mitochondria was resuspended with mitochondrial isolation buffer and the procedure was repeated twice. The combined supernatant was centrifuged at 37,500g for 4 minutes and the resulting pellet was homogenized with 1.0mL of mitochondrial isolation buffer using a glass/glass pestle by applying 10 strokes. After centrifugating at 5600g for 45 seconds, the supernatant was centrifuged again at 16,100g for 15 minutes resulting in the pellet containing the mitochondria. This pellet was stored at -80°C until further analysis.

For BN-PAGE analysis, the mitochondrial pellets were resuspended in 130-170µL of 750mM aminocaproic acid, 50mM Bis-Tris/HCl (pH 7.0) and 1.1% laurylmaltoside. After centrifugation at 16,100g for 15 minutes, the supernatants containing the oxidative phosphorylation enzyme complexes were kept on ice. Protein concentration of the mitochondrial proteins was measured using the Pierce Coomassie (Bradford) Protein assay Kit (Thermo Fisher Scientific cat. 23200).

Prior to loading on the Blue Native gel, a loading dye was added to the mixture and 5μL of 5% Serva Blue G and 750mM aminocaproic acid solution was added for each 100μL of solubilized protein solution with subsequently vortexing. Electrophoresis is first performed with the colored cathodal buffer (50mM Tris and 15mM Bis-Tris and 0.02% Serva Blue G) and anodal (25mM Bis-Tris) buffer at 80V for 1.5h at 4°C, until the dye-front reaches the resolving gel. Then, the colored cathodal buffer is replaced by the non-colored cathodal buffer (50mM Tris and 15mM Bis-Tris) and run at 200V for 2-3h at 4°C, until the Serva Blue tracking dye runs off the gel. Gels are stored between glass plates at -80°C until in-gel activity staining.

Samples were run in duplicate on two separate BN PAGE gels. The first gel was divided in three pieces: the upper, middle and lower part was used to evaluate complex I, complex III and complex IV activity, respectively. The second gel was divided in two pieces: the upper and lower part to evaluate the complex V and complex II activity, respectively. Complex I enzyme activity was evaluated after incubation of the gel part in a medium containing 2mM Tris-HCl 0.3mM NADH, 3.5mM NBT (pH 7.4) at 37°C for 3-4h. Complex II enzyme activity was evaluated after incubation in a medium containing 4.5mM EDTA, 10mM KCN, 0.2 mM phenazine methosulfate, 84mM sodium succinate and 10mM Nitro Blue tetrazolium chloride in 1.5mM phosphate buffer (pH 7.4) at 37°C for 3-4h. Staining of complex III was obtained by incubation with the 1-Step_TMB-Blotting Substrate Solution (Thermo Fisher Scientific) at 37°C, with maximum intensity of the bands after 5-6h. Staining of complex IV was obtained by incubation in 50mM phosphate buffer (pH 7.4), containing 2.3mM 3,3'-diaminobenzidine, 2mg/mL catalase, 1mg/mL cytochrome c and 220mM sucrose (pH 7.4) at 37°C, with maximum intensity of the bands after 5-6h. The activity of complex V was evaluated after incubation in 35mM Tris, 270mM glycine, 14mM $MgSO_4$, 0.2%

$Pb(NO_3)_2$, and 8mM ATP (pH 7.8) at 37°C, with maximum intensity of the bands after 2-3h. Gels were scanned using an Epson Perfection V800 Photo Scanner with Silverfast software in reflection mode (complex V) and in transmission mode (complex I-IV).

Mitochondrial DNA copy number

After homogenization of full liver tissue using the TissueLyser as described above, total DNA was isolated using DNeasy blood and tissue kit (Qiagen) according to the manufacturer's instructions. DNA concentrations were measured by spectrophotometry (Nanodrop ND-1000; Thermo Fisher Scientific). Analysis was performed on the LightCycler 480 (Roche) using DNA, SYBR Green mix (sensimix; Bioline) and specific mouse primers (Bioline) (Table S2).

Patients

Serum samples were collected from adolescents with severe obesity residing at the Zeepreventorium De Haan, and from lean controls. An informed consent was signed by all patients, controls and parents before enrollment. The study was approved by the ethical committee of Ghent University Hospital (BS-05660). Patients and controls underwent anthropometric measurements, serum biochemical analysis, ultrasound and FibroScan.

Liver steatosis and fibrosis were evaluated using ultrasound and FibroScan with controlled attenuation parameter (CAP) and liver stiffness measurement (LSM). Both were performed by the same experienced operator (S.L.) as described previously[1]. The FibroScan Mini+ 430 (Echosens, Paris, France) was used with the M- or XL-probe according to body size and the suggestion by the device. LSM ≥7kPa was considered

suggestive for liver fibrosis[2]. CAP values ≥248 dB/m were considered suggestive of steatosis. CAP values were classified as low (<248 dB/m), intermediate (248-300 dB/m), and high (>300 dB/m). In addition, liver steatosis was semi-quantitatively assessed on ultrasound with an LogiQ S7 device with a C1-6-D probe (GE Healthcare, Diegem, Belgium) or the Butterfly IQ (Butterfly Network, New York, NY). Liver steatosis was classified as absent, mild and moderate/severe, based on liver-kidney contrast, liver echogenicity, and visibility of the intrahepatic vessels and diaphragm[3].

Blood samples were obtained after overnight fasting. Serum triglycerides, glucose, insulin and alanine aminotransferase (ALT) were determined. Serum fibroblast growth factor 21 (FGF21) and mitochondrial open reading frame of the 12S rRNA-c (MOTS-C) were determined using enzyme-linked immunosorbent assay (DF2100; R&D systems and ELK7627; ELK Biotechnology, respectively). Homeostatic model assessment for insulin resistance (HOMA-IR) was calculated as the product of fasting insulin (mIU/L) x glucose (mg/dL)/405.

Supplementary tables

Supplementary table 1. Genes and primers used for qPCR on murine liver samples.

Gene	Abbrevation	Forward primer	Reverse primer
Hydroxymethylbilane synthase	Hmbs	AAGGGCTTTTCTGAGGCACC	AGTTGCCCATCTTTCATCACTG
Hypoxanthine guanine phosphoribosyl transferase	Hprt	GTTAAGCAGTACAGCCCCAAA	AGGGCATATCCAACAACAAACTT
Glyceraldehyde 3 phosphate dehydrogenase	Gapdh	CATGGCCTTCCGTGTTCCTA	GCGGCACGTCAGATCCA
Sterol regulatory element binding protein 1	Srebp1c	TGACCCGGCTATTCCGTGA	CTGGGCTGAGCAATACAGTTC
Carnitine palmitoyltransferase 1a	Cpt1a	CTCCGCCTGAGCCATGAAG	CACCAGTGATGATGCCATTCT
Tumor necrosis factor alpha	Tnfalpha	CATCTTCTCAAAATTCGAGTGACAA	TGGGAGTAGACAAGGTACAACCC
Collagen type I alpha 1	Col1a1	GCTCCTCTTAGGGGCCACT	CCACGTCTCACCATTGGGG
NADH dehydrogenase 1	Nd1	TGCACCTACCCTATCACTC	ATTGTTTGGGCTACGGCTC
Cytochrome b	Cytb	TACCTGCCCCATCCAACATT	TAAGCCTCGTCCGACATGAA
Cytochrome c oxidase I	Co1	ACCCAGATGCTTACACCACA	TGTGATATGGTGGAGGGCAG
ATP synthase 6	Atp6	CCACACACCAAAAGGACGAA	GAAGGAAGTGGGCAAGTGAG
peroxisome proliferative activated receptor gamma coactivator 1 alpha	Pgc1alpha	TCTCAGTAAGGGGCTGGTTG	TGACGCCAGTCAAGCTTTTTC
Nuclear respiratory factor 1	Nrf1	TATGGCGGAAGTAATGAAAGACG	CAACGTAAGCTCTGCCTTGTT
Transcription factor A	Tfam	GGAATGTGGAGCGTGCTAAAA	ACAAGACTGATAGACGAGGGG
OPA1 mitochondrial dynamin like GTPase	Opa1	TGGAAAATGGTTCGAGAGTCAG	CATTCCGTCTCTAGGTTAAAGCG
Mitofusin 1	Mfn1	CCTACTGCTCCTTCTAACCCA	AGGGACGCCAATCCTGTGA
Dynamin 1-like	Dnm1l	TTACGGTTCCCTAAACTTCACG	GTCACGGGCAACCTTTTACGA
Dynamin 2	Dnm2	TTTGGCGTTCGAGGCCATT	CAGGTCCACGCATTTCAGAC
Fission, mitochondrial 1	Fis1	AGGCTCTAAAGTATGTGCGAGG	GGCCTTATCAATCAGGCGTTC
BCL-2 interacting protein 3	Bnip3	TCCTGGGTAGAACTGCACTTC	GCTGGGCATCCAACAGTATTT
Parkin	Parkin	GAGGTCCAGCAGTTAAACCCA	CACACTGAACTCGGAGCTTTC

Supplementary table 2. Genes and primers used for determination of mitochondrial DNA copy number by RT-qPCR.

Gene	Forward primer	Reverse primer
D-loop	AGGCATGAAAGGACAGCA	TTGGCATTAAGAGGAGGG
18S rRNA	GAGAAACGGCTACCACATCC	CACCAGACTTGCCCTCCA

Supplementary figures

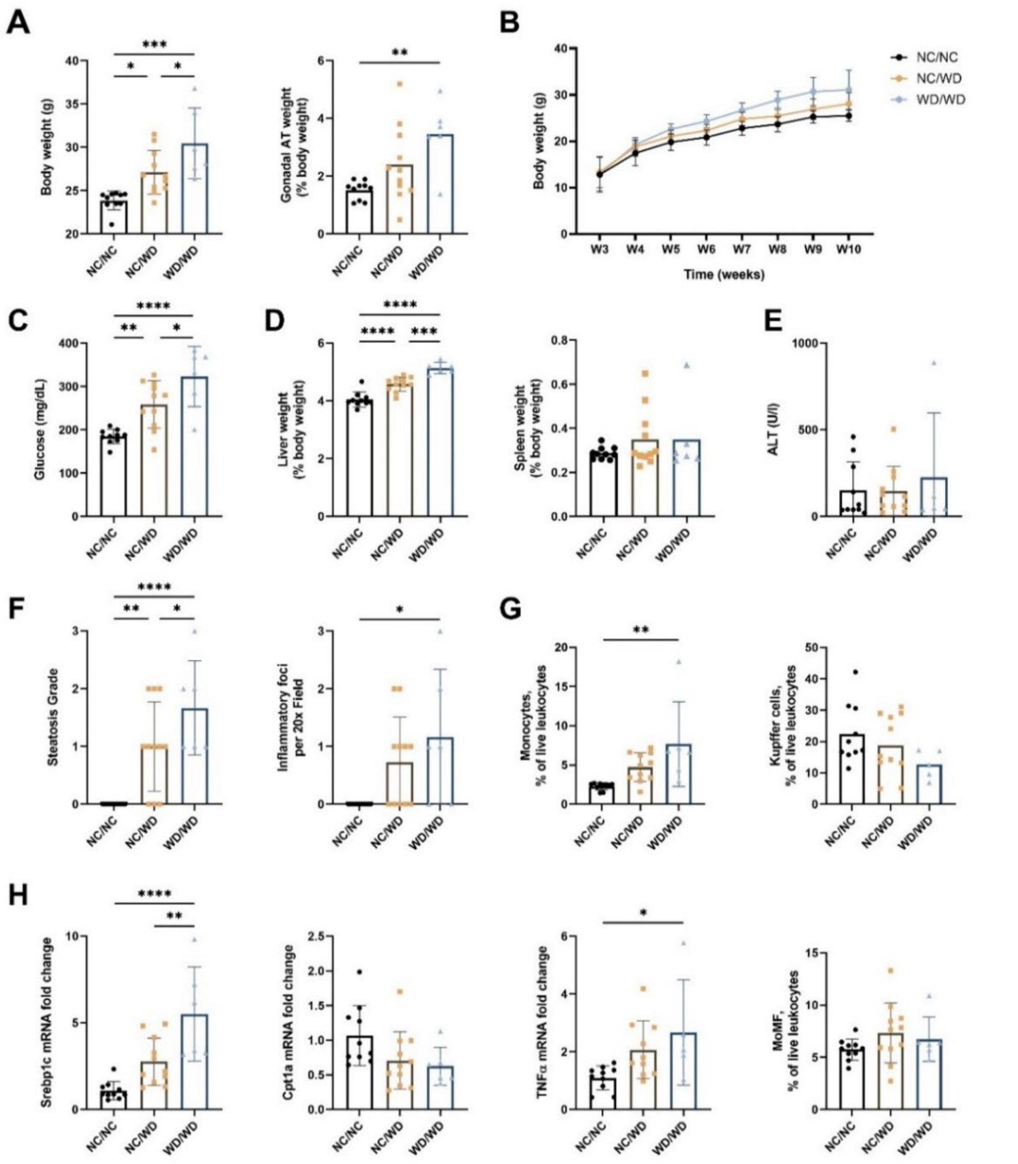

Fig. S1. Effect of maternal WD on 10-week-old male offspring. Body weight and relative gonadal adipose tissue weight (A). Body weight evolution (B). Serum glucose level (C). Relative liver and spleen weight (D). Serum ALT levels (E). Scoring of steatosis grade and inflammatory cell infiltration (F). Relative cell quantification of monocytes, KCs and MoMFs (G). Relative gene expression of *Srebp1c*, *Cpt1a* and *Tnfα* (H). Data are presented as mean ± SD. Statistical significance was evaluated by one-way ANOVA followed by Tukey post-hoc testing. * P<0.05; ** P<0.01; *** P<0.001; **** P<0.0001.

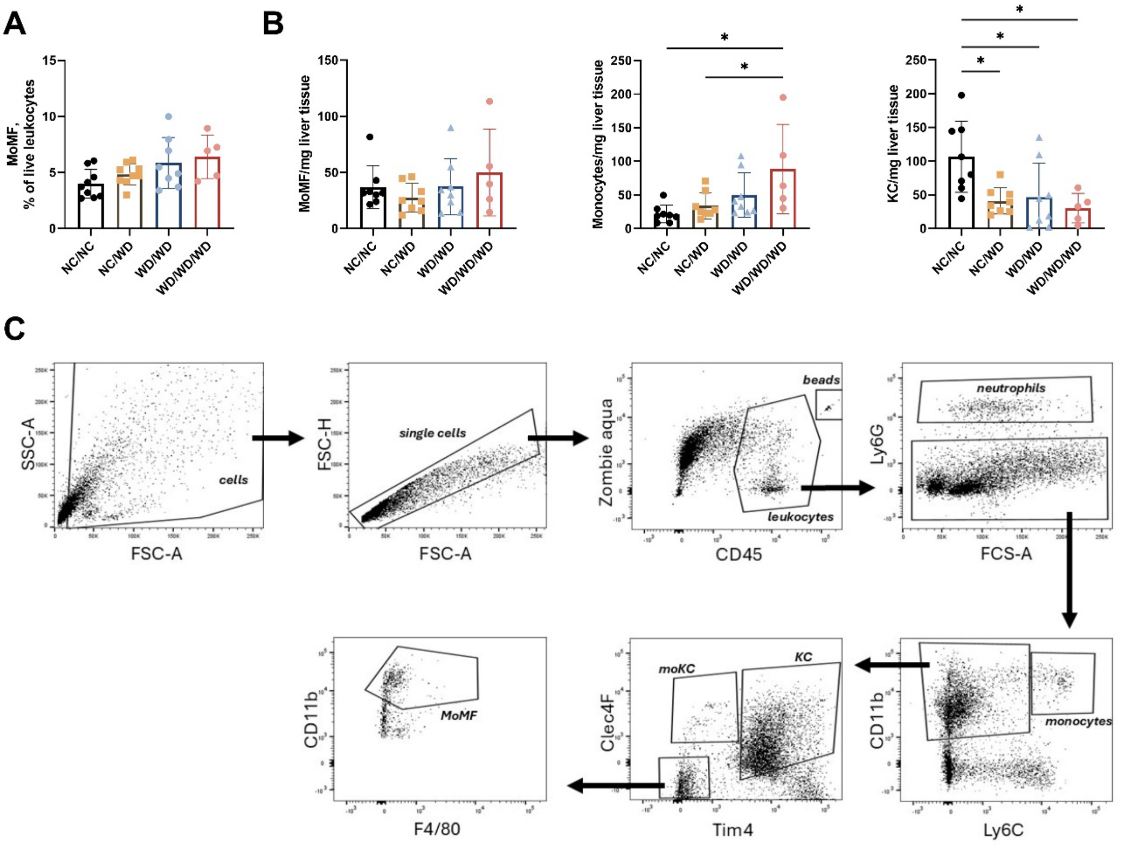

Fig. S2, related to figure 2. Effect of (multigenerational) maternal WD on MASLD development in male offspring at 16 and 3 weeks of age. Relative cell quantification of monocyte-derived macrophages (MoMF) (A). Absolute cell quantification per liver tissue weight of MoMF, monocytes and KCs (B). Gating strategy for flow cytometry analysis of the liver (C). Data are presented as mean ± SD. Statistical significance was evaluated by one-way ANOVA followed by Tukey post-hoc testing. * P<0.05.

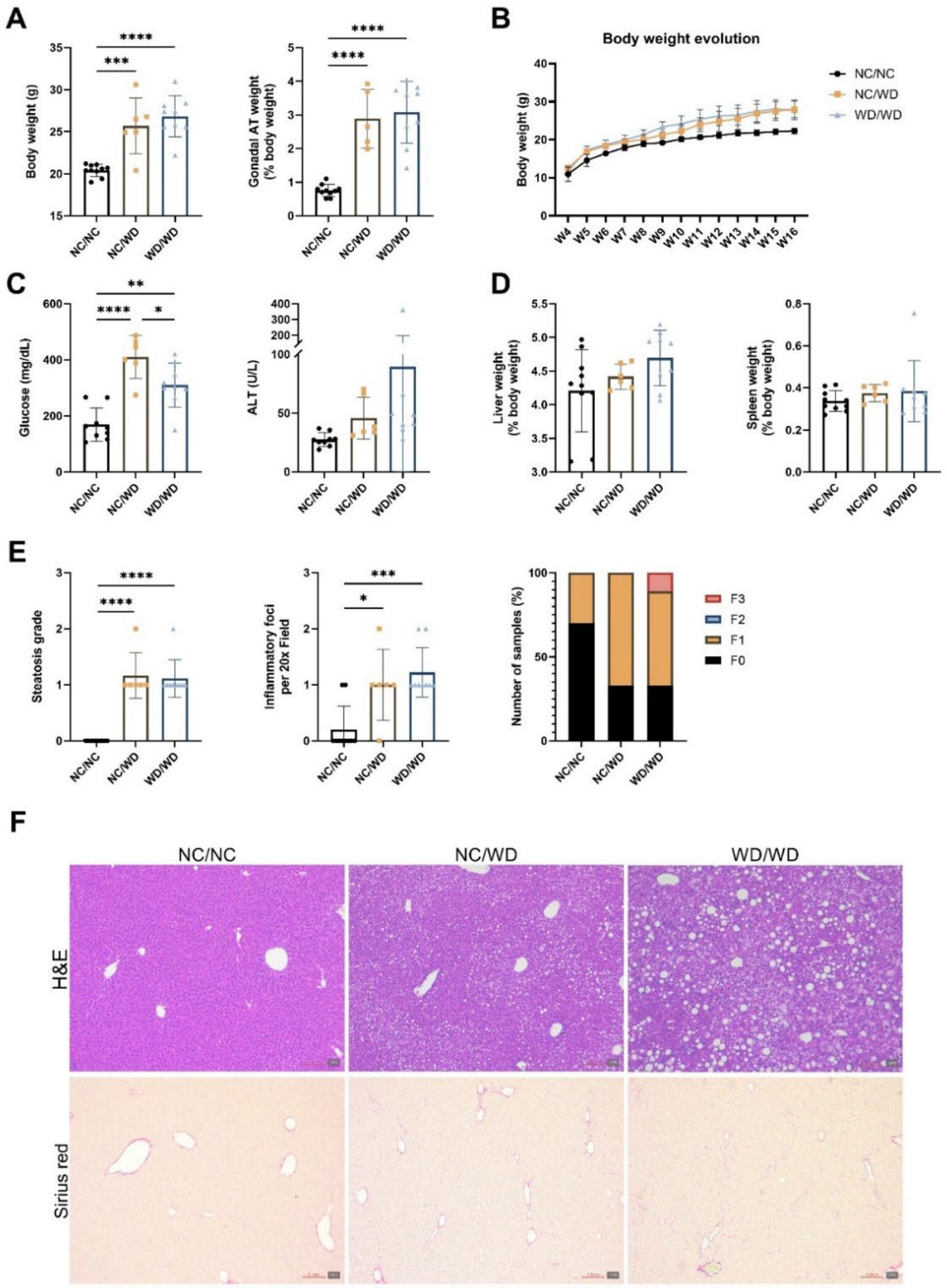

Fig. S3. Effect of maternal WD on female 16-week-old offspring. Body weight and relative gonadal adipose tissue weight (A). Body weight evolution (B). Serum glucose and ALT levels (C). Relative liver and spleen weight (D). Scoring of steatosis grade, inflammatory cell infiltration and fibrosis (E). Representative images of H&E and Sirius red stained slides (scale bar = 100μm) (F). Data are presented as mean ± SD. Statistical significance was evaluated by one-way ANOVA followed by Tukey post-hoc testing. * P<0.05; ** P<0.01; *** P<0.001; **** P<0.0001.

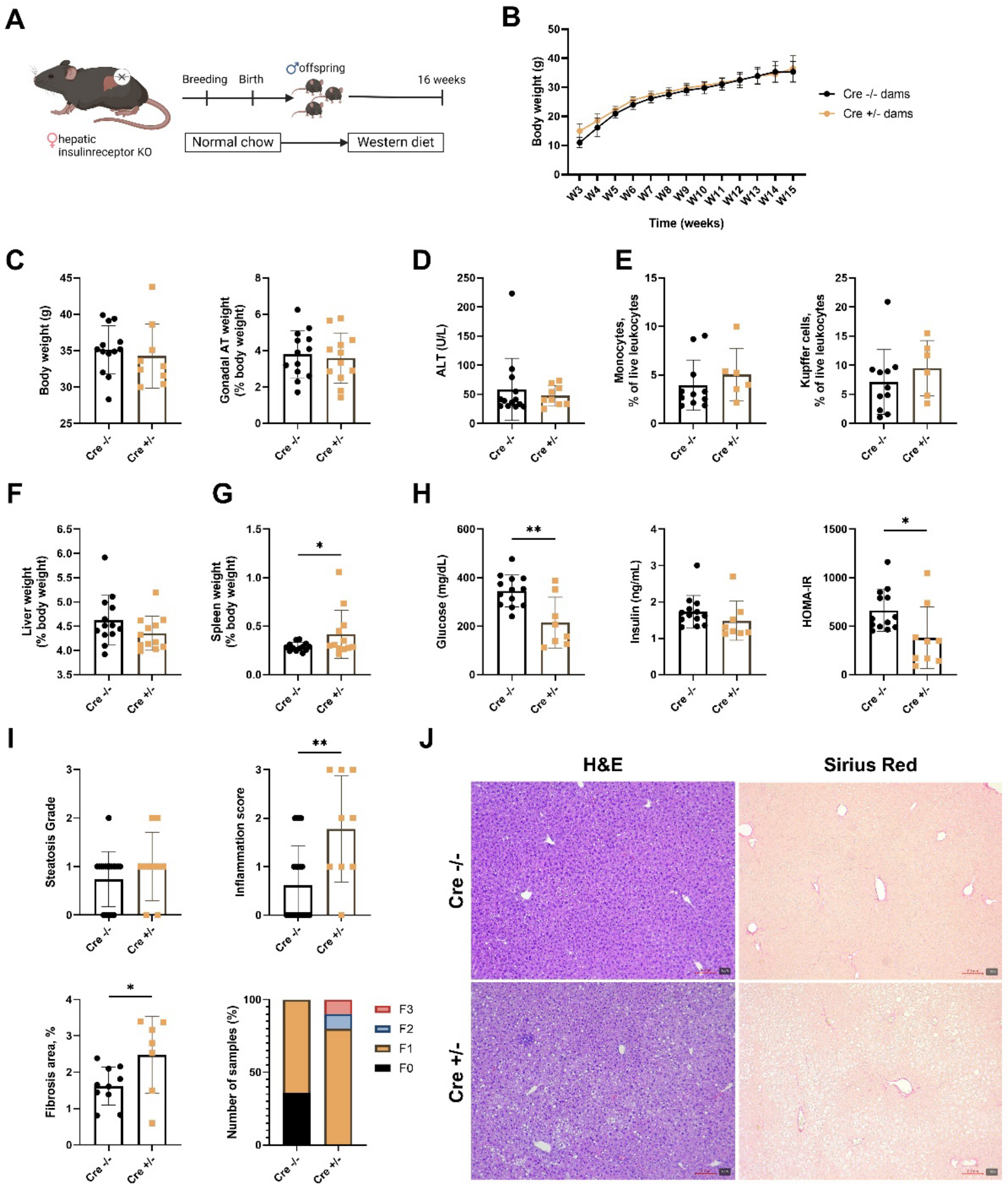

Fig. S4. Effect of maternal hepatic insulin resistance on male offspring. Schematic overview of the model. Created with BioRender (A). Body weight evolution (B), body and gonadal AT weight (C) and serum ALT levels (D) of 16-week-old offspring. Relative cell quantification of monocytes and KCs (E). Relative liver (F) and spleen weight (G). Serum glucose and insulin levels and HOMA-IR (H). Scoring of steatosis grade, inflammation and fibrosis and quantification of Sirius red area (I). Representative images of H&E and Sirius red stained slides (scale bars = 100µm) (J). Data are presented as mean ± SD. Statistical significance was evaluated by the unpaired student t-test. * P<0.05; ** P<0.01.

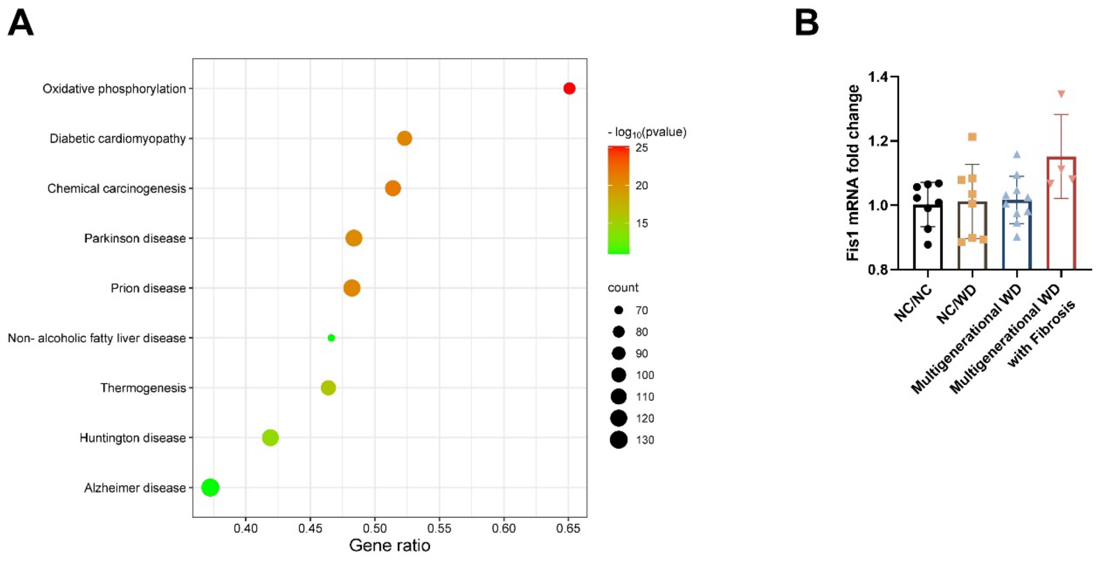

Fig. S5, related to figure 3. Effect of maternal WD on liver transcriptomics of 16-week-old offspring. Differentially regulated pathways between NC/WD and multigenerational WD + fibrosis after KEGG pathway analysis, plotted with p-value (shading), number of differentially regulated genes (circle size) and percentage of differentially regulated genes (x-axis) (A). Relative gene expression of *Fis1* (mitochondrial fission) (B). Data are presented as mean ± SD. Statistical significance was evaluated by one-way ANOVA followed by Tukey post-hoc testing.

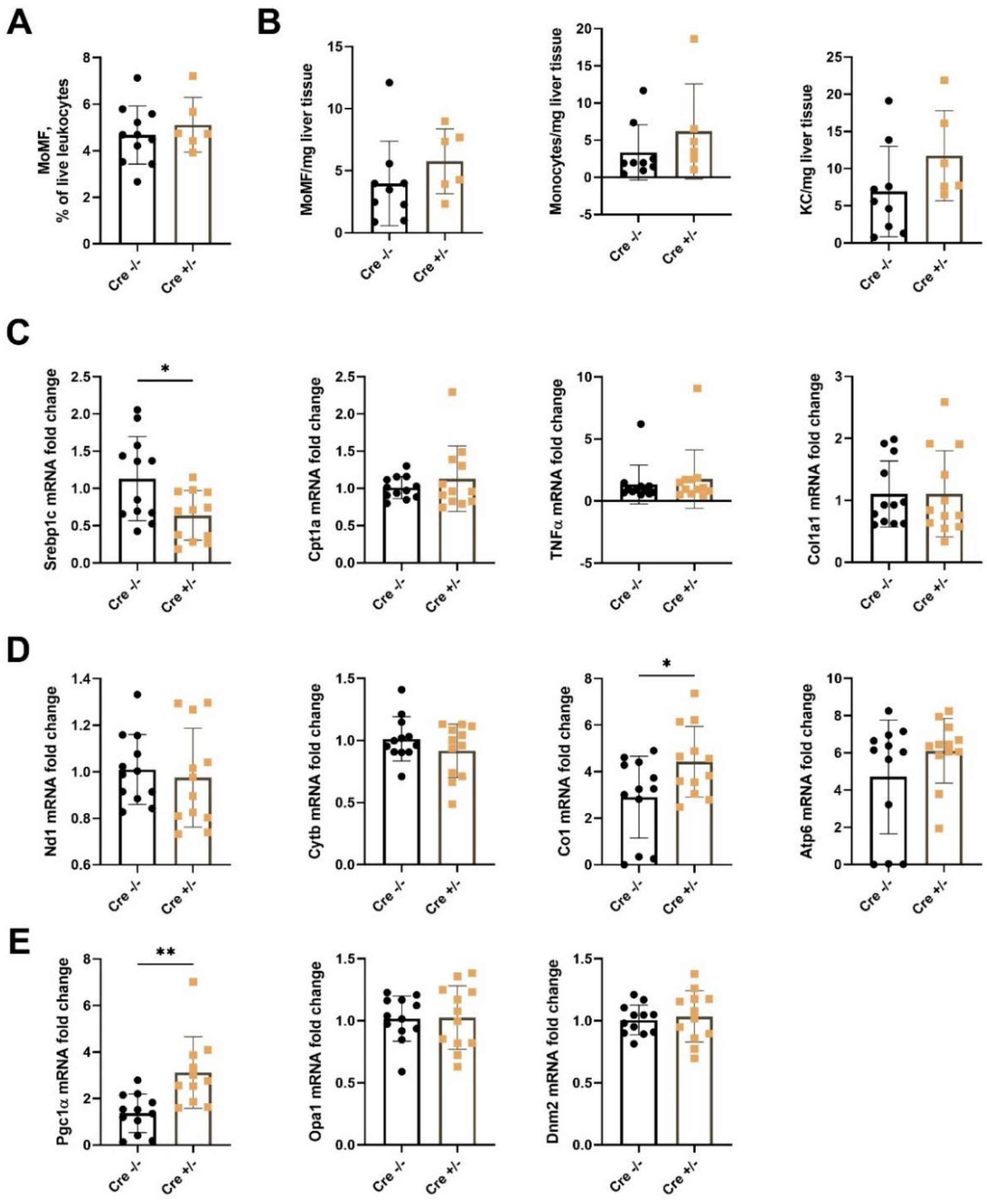

Fig. S6, related to Fig. S4. Effect of maternal hepatic insulin resistance on male offspring. Relative cell quantification of MoMFs (A). Absolute cell quantification per liver tissue weight of MoMFs, monocytes and KCs (B). Relative gene expression of *Srebp1c*, *Cpt1a*, *Tnfα* and *Col1a1* (C). Relative gene expression levels of mitochondrial-encoded OXPHOS subunits (*Nd1*, *Cytb*, *Co1*, and *Atp6*) (D). Relative gene expression levels of *Pgc1alpha*, *Opa1*, and *Dnm2* (E). Data are presented as mean ± SD. Statistical significance was evaluated by the unpaired student t-test. * P<0.05; ** P<0.01; *** P<0.001; **** P<0.0001.

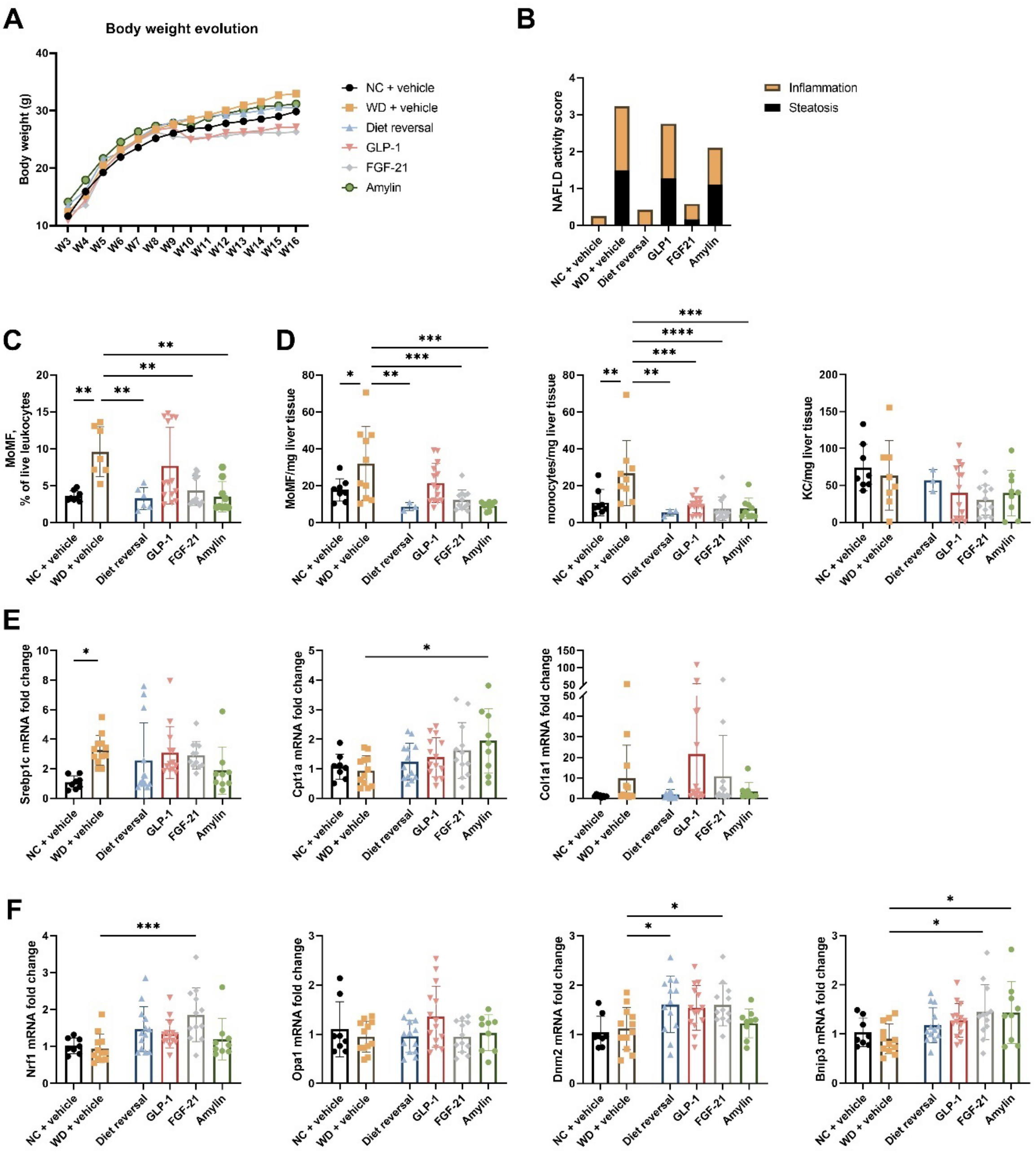

Fig. S7 related to figure 6. Evaluation of pharmacological compounds in the maternal WD model. Body weight evolution (A). MASLD activity score (B). Relative cell quantification of MoMFs (C). Absolute cell quantification per liver tissue weight of MoMFs, monocytes and KCs (D). Relative gene expression of *Srebp1c*, *Cpt1a*, *Tnfα* and *Col1a1* (E). Relative gene expression of *Nrf1*, *Opa1*, *Dnm2,* and *Bnip3* (F). Data are presented as mean ± SD. Statistical significance was evaluated by one-way ANOVA followed by Tukey post-hoc testing. * P<0.05; ** P<0.01; *** P<0.001; **** P<0.0001.

Supplementary references

[1] Lefere S, Dupont E, De Guchtenaere A, Van Biervliet S, Vande Velde S, Verhelst X, et al. Intensive Lifestyle Management Improves Steatosis and Fibrosis in Pediatric Nonalcoholic Fatty Liver Disease. Clinical Gastroenterology and Hepatology 2022;20:2317-2326.e4. https://doi.org/10.1016/j.cgh.2021.11.039.

[2] Nobili V, Vizzutti F, Arena U, Abraldes JG, Marra F, Pietrobattista A, et al. Accuracy and reproducibility of transient elastography for the diagnosis of fibrosis in pediatric nonalcoholic steatohepatitis. Hepatology 2008;48:442–8. https://doi.org/10.1002/hep.22376.

[3] Shannon A, Alkhouri N, Carter-Kent C, Monti L, Devito R, Lopez R, et al. Ultrasonographic quantitative estimation of hepatic steatosis in children With NAFLD. J Pediatr Gastroenterol Nutr 2011;53:190–5. https://doi.org/10.1097/MPG.0b013e31821b4b61.
